# Epigenetics and the city: Non‐parallel DNA methylation modifications across pairs of urban‐forest Great tit populations

**DOI:** 10.1111/eva.13334

**Published:** 2022-01-04

**Authors:** Aude E. Caizergues, Jeremy Le Luyer, Arnaud Grégoire, Marta Szulkin, Juan‐Carlos Senar, Anne Charmantier, Charles Perrier

**Affiliations:** ^1^ CEFE, Univ Montpellier, CNRS, EPHE, IRD Montpellier France; ^2^ Ifremer, IRD, Institut Louis‐Malardé Univ Polynésie Française, EIO Taravao French Polynesia; ^3^ Centre of New Technologies University of Warsaw Warsaw Poland; ^4^ Museu de Ciències Naturals de Barcelona Barcelona Spain; ^5^ CBGP, INRAe, CIRAD, IRD, Montpellier SupAgro Univ. Montpellier Montpellier France

**Keywords:** adaptation, DNA methylation, epigenomics, genomics, urbanization

## Abstract

Identifying the molecular mechanisms involved in rapid adaptation to novel environments and determining their predictability are central questions in evolutionary biology and pressing issues due to rapid global changes. Complementary to genetic responses to selection, faster epigenetic variations such as modifications of DNA methylation may play a substantial role in rapid adaptation. In the context of rampant urbanization, joint examinations of genomic and epigenomic mechanisms are still lacking. Here, we investigated genomic (SNP) and epigenomic (CpG methylation) responses to urban life in a passerine bird, the Great tit (*Parus major*). To test whether urban evolution is predictable (i.e. parallel) or involves mostly nonparallel molecular processes among cities, we analysed both SNP and CpG methylation variations across three distinct pairs of city and forest Great tit populations in Europe. Our analyses reveal a polygenic response to urban life, with both many genes putatively under weak divergent selection and multiple differentially methylated regions (DMRs) between forest and city great tits. DMRs mainly overlapped transcription start sites and promotor regions, suggesting their importance in modulating gene expression. Both genomic and epigenomic outliers were found in genomic regions enriched for genes with biological functions related to the nervous system, immunity, or behavioural, hormonal and stress responses. Interestingly, comparisons across the three pairs of city‐forest populations suggested little parallelism in both genetic and epigenetic responses. Our results confirm, at both the genetic and epigenetic levels, hypotheses of polygenic and largely nonparallel mechanisms of rapid adaptation in novel environments such as urbanized areas.

## 1 INTRODUCTION

Identifying mechanisms involved in rapid adaptation to novel environmental conditions is a central theme in evolutionary biology and a pressing concern in the context of global changes characterizing the Anthropocene (Malhi, 2017). The vast majority of studies investigating mechanisms involved in rapid adaptation to new environments have focused on phenotypic plasticity on the one hand and on genetic responses to selection on the other hand. At their crossroad, recent work underlines the potential role of epigenetics in rapid adaptation to new environments (Liu, 2013). In particular, environmental variations can induce differences in DNA methylation patterns and hence modulate gene expression and upper‐level phenotypes (Duncan et al., 2014; Jaenisch & Bird, 2003). Such methylation‐linked phenotypic variation can occur during an individual's lifetime, especially early on during the organism's development (Waterland & Jirtle, 2003; Weaver et al., 2004). Although methylation changes acquired across an individual's lifetime may often be nonheritable (Reik et al., 2001) but see (Crews et al., 2007; Janowitz Koch et al., 2016), epigenetically induced phenotypic shifts may nevertheless enhance individual fitness in new environments. Moreover, during the course of evolution, divergent genetic variants regulating epigenetic modifications may also be under selection, hence promoting the evolution of divergent epigenotypes and epigenetically linked phenotypic variation (Richards et al., 2010). While epigenetic studies focused on human diseases and medical topics are now abundant, studies in an ecological context are still rare (Derks et al., 2016). A few epigenetic studies in natural plant populations revealed that DNA methylation shifts might play a determinant role in local adaptation to environmental variation (Dubin et al., 2015; Foust et al., 2016), however regulation and effects of DNA methylation are quite different between plants and vertebrates and methylation studies in vertebrates are rare (McNew et al., 2017). There is hence an urgent need for further empirical investigations of simultaneously rapid genetic and epigenetic evolution in response to environmental change (Danchin et al., 2011).

Urbanization rapidly and irreversibly changes natural habitats into human‐made environments and is considered a major threat to biodiversity (Brondizio et al., 2019). For species who appear to cope with urbanization, urban habitats present a myriad of novel environmental conditions compared to the habitat where they evolved, including high levels of chemical, light and sound pollution, high proportion of impervious surfaces, high habitat fragmentation, low vegetation cover and high human densities (Grimm et al., 2008; Szulkin, Garroway, et al., 2020; Szulkin, Munshi‐South, et al., 2020). Such extreme environmental changes compared to natural areas are expected to result in numerous novel selection pressures for city‐dwelling species (Szulkin, Garroway, et al., 2020; Szulkin, Munshi‐South, et al., 2020). Accordingly, rates of recent phenotypic change, concerning multiple types of traits related to behaviour, morphology, phenology and physiology, were found greater in urban areas than in any other habitat types, including nonurban anthropogenic contexts (Alberti et al., 2017; Thompson et al., 2018). The exploration of the molecular mechanisms implicated in urban‐driven phenotypic changes has only begun, with both genetic (Mueller et al., 2013; Perrier et al., 2018; Salmón et al., 2021), and epigenetic investigations (McNew et al., 2017; Riyahi et al., 2015; Watson et al., 2021). For instance, DNA methylation variations have been associated in vertebrates with high levels of traffic‐related air pollution (Ding et al., 2017). Yet, epigenetic studies have been performed at relatively small genomic resolution. In addition, very little is known about the level of parallelism and hence of the predictability of genetic and epigenetic evolution in response to urbanization in distinct cities (Rivkin et al., 2018; Santangelo et al., 2018). So far, a small number of studies have provided evidence for a range of situations: from local adaptation despite strong gene flow (e.g. in the Great tit: Salmón et al., 2021; in the red‐tailed bumblebee *Bombus lapidaries*: Theodorou et al., 2018) to restricted gene flow and independent colonization in different cities by a few founders, followed by adaptation (e.g. in the burrowing owl *Athene cunicularia*, Mueller et al., 2018). Providentially, recent genomic tools of high resolution and the multitude of cities around the globe offer unique opportunities to compare simultaneously individuals’ genomic and epigenomic responses in several cities and thereby study the parallelism and predictability in molecular mechanisms implicated in rapid adaptation to urbanization (Perrier et al., 2020; Santangelo et al., 2020).

In this study, we used both genome‐wide and epigenome‐wide sequencing approaches to compare genetic and epigenetic responses among three pairs of great tit *Parus major* populations in urban and forest habitats. At the European level, population monitoring of Great tits revealed parallel phenotypic shifts in city birds compared to their forest conspecifics, with in particular smaller and lighter urban birds laying earlier and smaller clutches (Biard et al., 2017; Caizergues et al., 2021; Chamberlain et al., 2009; Corsini et al., 2020). In addition, genomic analyses showed patterns of genome‐wide differentiation between urban and forest birds (Perrier et al., 2018) while a large‐scale analysis revealed some parallel footprints of adaptation to urbanization across nine European cities (Salmón et al., 2021). At the epigenetic level, a preliminary Great tit study recently described methylation shifts associated with urbanization (Watson et al., 2021). However, this analysis focused on a single location (*n* = 6 urban and 6 forest males) and hence could not test for a potential parallelism in the urban‐related epigenomic response. In order to advance our understanding of the genome‐wide and epigenome‐wide responses to urbanization, and its putative spatial parallelism, we here searched for genomic footprints of divergent selection and for DMRs between three pairs of urban‐forest populations across Europe, thereby complementing the work of Salmón et al. (2021) with an epigenomic insight into great tit adaptation to urbanization. Our results show that despite limited genetic differentiation and few genomic footprints of divergent selection between forest and urban populations, urban life was associated with numerous differentially methylated regions notably associated with neural development, behaviour and immunity. Hence, this study suggests that shifts in DNA methylation patterns could play a role in adaptation to urbanization. Importantly, we found little parallelism between cities in both the genomic and the epigenomic responses to urbanization, possibly confirming the hypothesis that multiple evolutionary ways exist to independently cope with similar novel environmental conditions.

## 2 MATERIAL AND METHODS

### 2.1 Study sites and sampling

**FIGURE 1:**
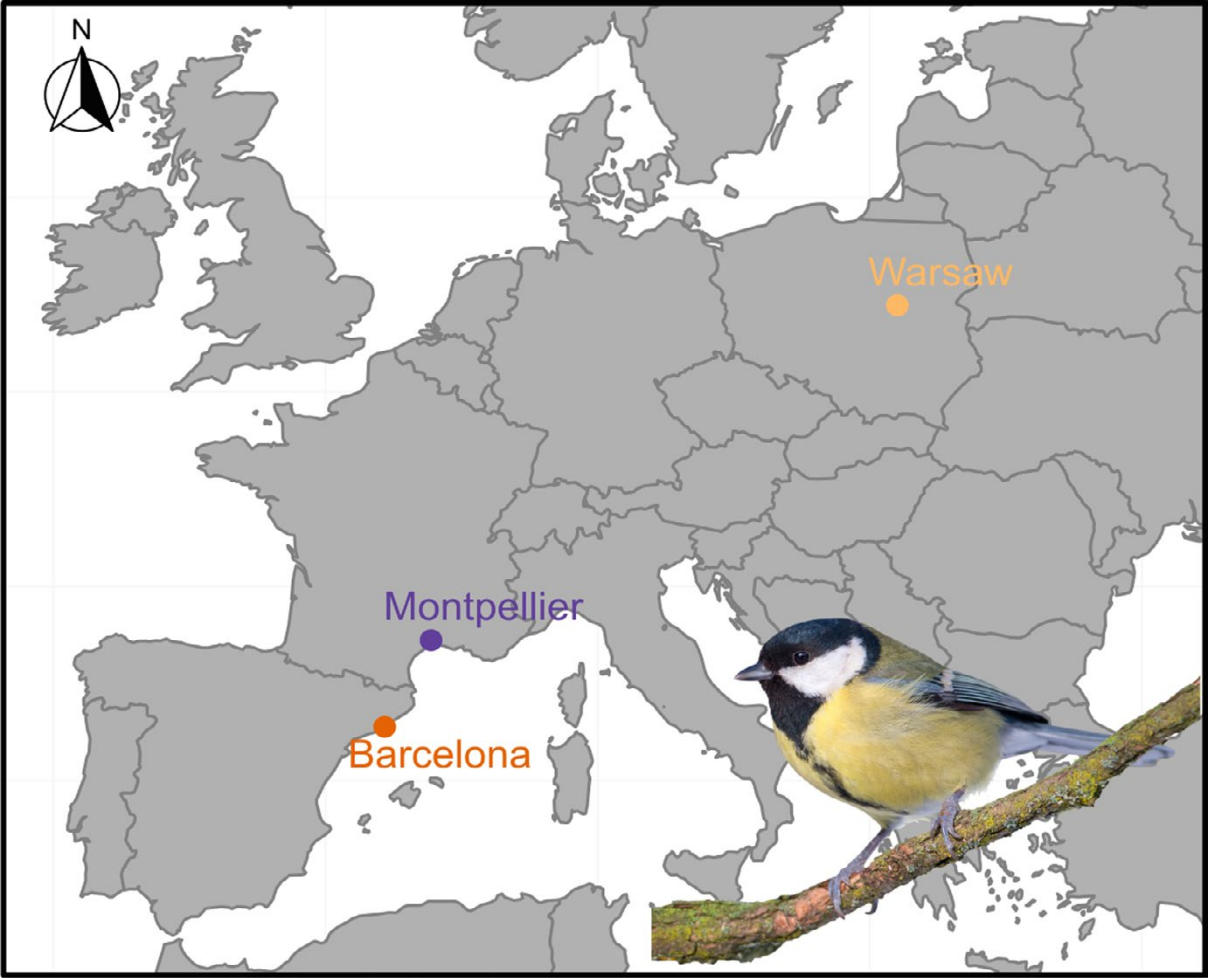
Great tit blood sample locations in Europe (in urban and forest sites in and near Barcelona, Montpellier & Warsaw)

Three pairs of great tit populations in urban and forest environments were sampled in the three European cities of Barcelona (Spain), Montpellier (France) and Warsaw (Poland), Figure 1. For each location, 10 individuals were sampled within the city and 10 individuals were sampled from nearby forest. Blood samples were collected from breeding individuals during spring between 2016 and 2018 (except 2 individuals for Barcelona city, collected in 2014 and 2015) and kept in 96% Ethanol or Queen's Lysis Buffer. Samples had balanced sex ratio (5 males and 5 females for each population) except for the forest population of Barcelona where 6 females and 4 males were sampled. The sample design did not include any closely related individuals (i.e. no full‐ or half‐sibling nor parents/offsprings): (1) we chose to sequence individuals that were not full‐sibs or half‐sibs, based on field pedigrees and (2) we subsequently confirmed that neither full‐sibs nor half‐sibs were present in the dataset, by inspecting identity‐by‐state measured between all pairs of individuals using the RADseq data.

### 2.2 DNA extraction, RAD‐seq and reduced‐representation bisulphite sequencing

We used QIAGEN DNeasy blood and tissue kits to extract DNA from blood samples, following the provided instructions for nucleated blood samples. DNA was quantified using a NanoDrop ND8000 spectrophotometer and a Qubit 2.0 fluorometer with the DNA HS assay kit (Life Technologies). DNA quality was examined on agarose gels. We then performed RAD‐sequencing and RRBS‐sequencing using standard protocols. For RAD‐sequencing (restriction‐site‐associated DNA sequencing, Baird et al., 2008), the library preparation was done by the Montpellier GenomiX (MGX) platform (CNRS, Montpellier), using the enzyme SbfI. Each individual was identified using a unique six nucleotides tag, individuals were randomly multiplexed in equimolar proportions by libraries of 37 individuals. Each library was sequenced on a lane of an Illumina HiSeq 2500. Paired‐end sequencing was used to produce 150 bp reads. This generated an average of 4.9M reads per individual. The DNA of the 60 individuals were processed twice to test for reliability of the genotyping process. The RRBS‐sequencing started with DNA digestion using MspI restriction enzymes, which cuts CCGG sites and target regions that are CG rich, permitting to have a high proportion of sequences in promotor regions. Individuals were randomly multiplexed in equimolar proportions by libraries of 10 individuals. Bisulphite treatment converted unmethylated cytosines into uracil, then converted to thymine after PCR amplification. Each library was then sequenced on a lane of an Illumina HiSeq 2500. Paired‐end sequencing was used to produce 50 bp reads. This generated an average of 19.3M reads per individual.

### 2.3 SNP calling and statistical genomic analyses

Fastp v. 0.19.7 (Chen et al., 2018) was used to trim the RAD‐seq reads, keeping reads with a minimum quality of 15 before mapping individual sequences against the reference genome of the Great tit (Laine et al., 2016; GenBank assembly accession: GCA_001522545.3) with BWA v0.7.17 (Li & Durbin, 2010). Genotyping was conducted with stacks v2.41 (Rochette et al., 2019) “*gstacks*” and “*population*” functions, using “*snp*” model, filtering for mapping quality >10, and alpha = 0.05. Still using the population module, we kept SNPs with minor allele frequency >0.01, observed heterozygosity <0.65, and genotyped in at least 90% of individuals in each population. We obtained 181,041 SNPs. Then, using vcftools v0.1.15 (Danecek et al., 2011), loci with minor allele frequency <0.05 were removed, shortening the dataset to 75,246 SNPs. Still using vcftools, SNPs with extremely low or high coverage (5%–95% of the distribution) were removed, resulting in 74,137 SNPs retained for subsequent population genomic analyses.

To document genomic variation among urban and forest great tits from the three locations we used a redundancy analysis (RDA), with location (Barcelona, Montpellier and Warsaw), environment (urban or forest) and sex as explanatory variables. Partial RDA was also produced to test for each variable effect (environment, location or sex) alone after controlling for all other variables. The effect of a given factor was considered significant with a *p*‐value < 0.05.

To estimate genome‐wide differentiation between populations we used Weir and Cockerham's *F*_ST_ (Weir & Cockerham, 1984) computed using the StAMPP R package (Pembleton et al., 2013). Average *F*_ST_ was estimated using all SNPs, and confidence intervals were assessed using 1000 bootstrap replicates.

We used two methods to investigate outlier SNPs potentially under divergent selection between forest and urban populations: an *F*_ST_‐outlier based method (using Bayescan v2.1, Foll & Gaggiotti, 2008), and a multivariate method (using a RDA, Forester et al., 2016) aiming respectively at identifying strong outliers indicating footprints of differentiation for each population pair and weaker outliers that represent footprints of divergent selection typically expected in polygenic adaptations in response to complex environmental heterogeneity across several population pairs studied at once.

First, we ran Bayescan (with default parameter options) for each pair of populations (Barcelona, Montpellier and Warsaw) separately to detect SNPs with outlier values of *F*_ST_. As recommended by Foll and Gaggiotti (2008) we considered SNPs as outliers when they displayed a *q*‐value above the 0.1 threshold. Second, following a similar procedure as described above for the RDA analysis, we used a constrained RDA to investigate the effect of habitat (forest vs. city) and to identify outliers SNPs that displayed more than 3 times SD from the mean score on the constrained axis (Forester et al., 2016).

### 2.4 Methylation calling and epigenomic statistical analyses

The RRBS reads were first trimmed using fastp software v0.19.7 (Chen et al., 2018), and quality filtered to keep only reads with a quality >15. The BISMARK software v0.20.0 (Krueger & Andrews, 2011) was used for mapping reads on the masked reference genome with default parameters and a maximum of one allowed mismatch (see Table S1 for mapping and filtration details). Note that BISMARK implements methylation calls for the entire R1 and for the R2 until reads overlap, to prevent double counts. Methylation information for cytosines in a CpG context with sufficient coverage (≥10×) was extracted.

Similarly to genetic differentiation, an RDA was performed to describe epigenomic variation (using level of methylation measured at each CpG position of the genome) across location, habitat and sex. A partial RDA was also conducted to test for the habitat effect alone. Additionally, we investigated more finely whether individual methylation on CpG cytosines varied across location, habitat (urban vs. forest), sex, and a location × habitat interaction, using an ANOVA, run on autosomes and Z chromosome separately.

We used the MethylKit R package to identify differentially methylated regions (DMRs) between groups of individuals (habitat or sex) for each location. With a logistic regression including sex as covariate (“calculateDiffMeth” function) on normalized coverage data (“normalizeCoverage” function), we looked for 1000 bp regions (produced by cutting the genome into 1000 bp units; mean ± SD = 33,682 ± 664 tiles per location; mean ± SD = 28.940 ± 17.906 CpG/tiles) differing by at least 10% (as usually reported in the literature) of methylation between urban and forest individuals (regions present in at least 9 over 10 individuals). Methylkit was used with the option destrand = TRUE, collapsing both strands because (1) it is recommended for studies that focus on CpG only and (2) do not explore hemimethylation. We kept only DMRs with a *q*‐value < 0.001, as suggested in Methylkit's vignette, that were considered as significantly different between habitats. Similarly, we ran a logistic regression to identify DMRs between sexes, including habitat as covariable.

### 2.5 Genes associated with SNP outliers and DMRs, and gene ontology analyses

We investigated whether genomic outliers and DMRs overlapped genes in 5 kb upstream and downstream regions using BEDTools v2.28.0 (Quinlan & Hall, 2010). Gene ontology analyses were performed with the R package topGO (Alexa & Rahnenführer, 2009), with GO referenced for the chicken *Gallus gallus* (ggallus_gene_ensembl) and using a background list of genes covered in our dataset, to identify potential statistically enriched genes ontologies among the lists of genes extracted. In order to take into account hierarchy and nonindependence in GO terms we used the “weight01” algorithm with a “fisher” statistic (options *algorithm*=*“weight01”* and *statistics*=*“fisher”* of the *runTest()* function). GO terms were considered significantly enriched when having a weightFisher <0.05.

## 3 RESULTS

### 3.1 Small genetic and epigenetic average differentiation between urban and forest populations

#### 3.1.1 Genetic differentiation

**FIGURE 2:**
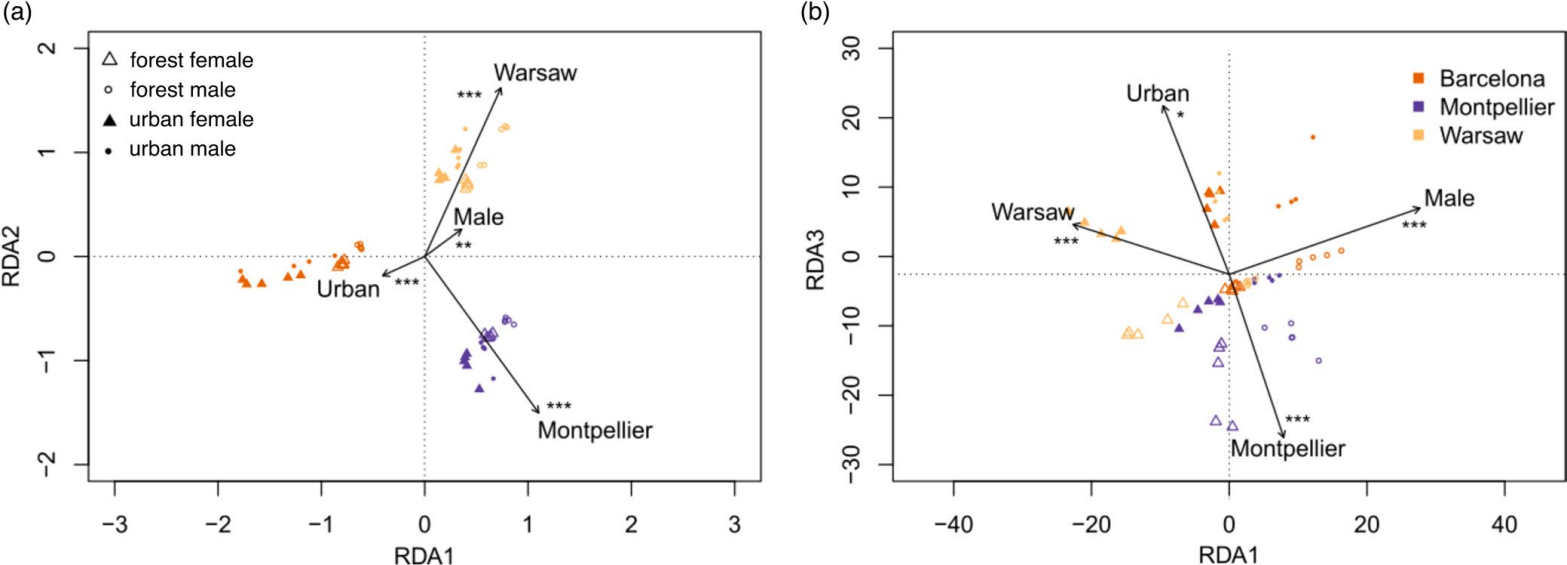
Rdancy analyses (RDA) on (a) genomic data (74,137 filtered SNPs) and (b) methylation levels (based on methylation levels observed at 157,741 positions). Triangles represent forest habitats, circles represent urban habitats, empty and solid symbols represent females and males respectively. ***p‐value < 0.001, **p‐value < 0.01 and *p‐value < 0.05, related to the explanatory factors

The redundancy analysis (RDA) performed on 74,137 SNPs obtained by RAD‐sequencing, including location (Barcelona, Montpellier or Warsaw), habitat (urban vs. forest), and sex as explanatory variables was highly significant (*p *< 0.001) but explained only a small fraction (i.e. less than 2%) of the total variance (*R* ^2^ = 0.018, Figure 2a, Table S2). All three variables were significant (location: *p* = 0.001; habitat: *p* = 0.004; sex: *p* = 0.001) revealing small genetic structuration between groups. Partial RDA revealed that the net variation explained by habitat (*R* ^2^ = 0.004, *p* = 0.001) was inferior to the net variation explained by location (*R* ^2^ = 0.012, *p* = 0.001) but higher than sex (*R* ^2^ = 0.002, *p* = 0.004, Table S2). As expected, when removing the Z chromosome from the data, sex became nonsignificant (*p* = 0.260), whereas the effects of other variables remained significant and of similar magnitude (Table S3).

Genome‐wide differentiation between populations was relatively low on average (mean *F*_ST_ = 0.019), in the order of 1%–2% between habitats for each location (*F*_ST Barcelona(forest‐urban)_ = 0.018 ± 0.001; *F*_ST Montpellier(forest‐urban)_ = 0.012 ± 0.001; *F*_ST Warsaw(forest‐urban)_ = 0.018 ± 0.001, Table 1), suggesting relatively high gene flow and limited genetic drift among populations. Mean *F*_ST_ on autosomes was lower (mean *F*_ST_ = 0.014, Table S4) than on the Z chromosome (mean *F*_ST_ = 0.022, Table S5).

**TABLE 1:** *F*_ST_ estimation between pairs of subpopulations

	Montpellier forest	Montpellier‐urban	Warsaw‐forest	Warsaw‐urban	Barcelona‐forest
Montpellier‐urban	0.012 (0.011–0.013)	–	–	–	–
Warsaw‐forest	0.009 (0.009–0.010)	0.018 (0.017–0.019)	–	–	–
Warsaw‐urban	0.018 (0.017–0.018)	0.026 (0.025–0.027)	0.018 (0.017–0.019)	–	–
Barcelona‐forest	0.009 (0.009–0.010)	0.019 (0.017–0.018)	0.009 (0.009–0.010)	0.018 (0.017–0.019)	–
Barcelona‐urban	0.025 (0.024–0.027)	0.034 (0.033–0.035)	0.025 (0.025–0.026)	0.034 (0.033–0.034)	0.018 (0.017–0.019)

Note

95% confidence intervals in brackets were computed using StAMPP package with 1000 bootstrap.

#### 3.1.2 Methylation variation

Similarly to genetic data, we performed an RDA on methylation level of 157,741 CpG sites to describe epigenetic variation among individuals in relation to location, habitat and sex (Figure 2b). The model was significant but explained less than 1% of the total variance (*R* ^2^ = 0.007, *p* = 0.001). All variables contributed significantly (location: *p* = 0.001, habitat: *p* = 0.03, sex: *p* = 0.001, Table S6). Partial RDA revealed that location and sex explained a similar proportion of the total variance which was higher than habitat (location: *R* ^2^ = 0.003, *p* = 0.001; sex: *R* ^2^ = 0.003, *p* = 0.001; habitat: *R* ^2^ = 0.001, *p* = 0.3). When removing the sex chromosome from analyses, results remained similar (Table S7), showing that the difference in methylation was not entirely driven by sexual chromosomes.

**FIGURE 3:**
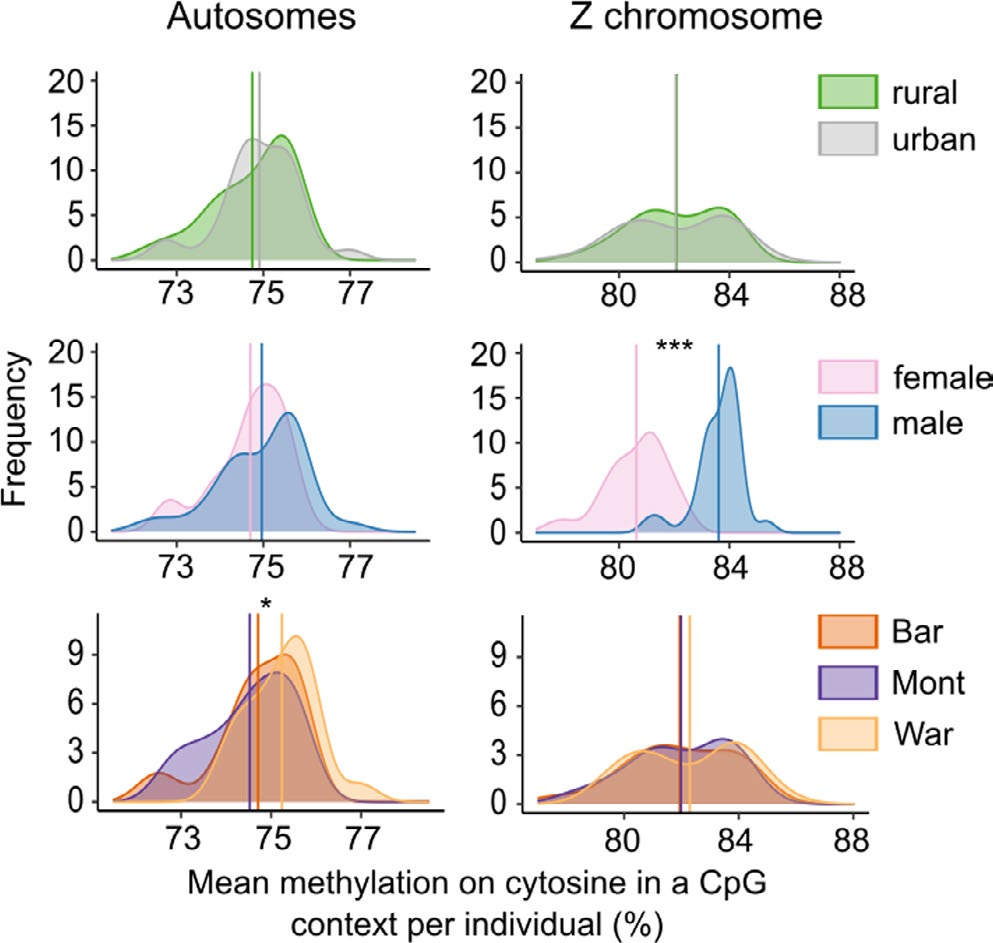
Distributions of mean per cent of methylation on autosomes and on the Z chromosome, compared between habitats (forest vs. urban), sexes and locations

We then investigated more finely whether individual methylation on CpG cytosines varied across location, habitat (urban vs. forest), sex, and location × habitat interaction, using an ANOVA, run on autosomes and Z chromosome separately (Figure 3, Table S8). For autosomes, we detected a significant effect of location (*F* = 3.319, *p* = 0.044), with Montpellier individuals showing lower methylation levels than Warsaw ones (Figure 3, Tukey test: *p* = 0.04) and no other difference between pairs of cities. Also, no significant effect of sex (*p* = 0.263) or habitat (*p* = 0.478) was found, suggesting that urbanization did not have an important overall effect on global methylation levels. For the Z chromosome, we found a strong difference between sexes, with homogametic males showing 2.98% more methylated Z than heterogametic females (Figure 3; *p* = 1.45 × 10^−15^), while no significant difference between location (*p* = 0.577) or habitat (*p* = 0.915) was found.

### 3.2 Nonparallel yet strong genomic footprints of divergent selection between urban and forest populations and evidence for polygenic adaptation

**FIGURE 4:**
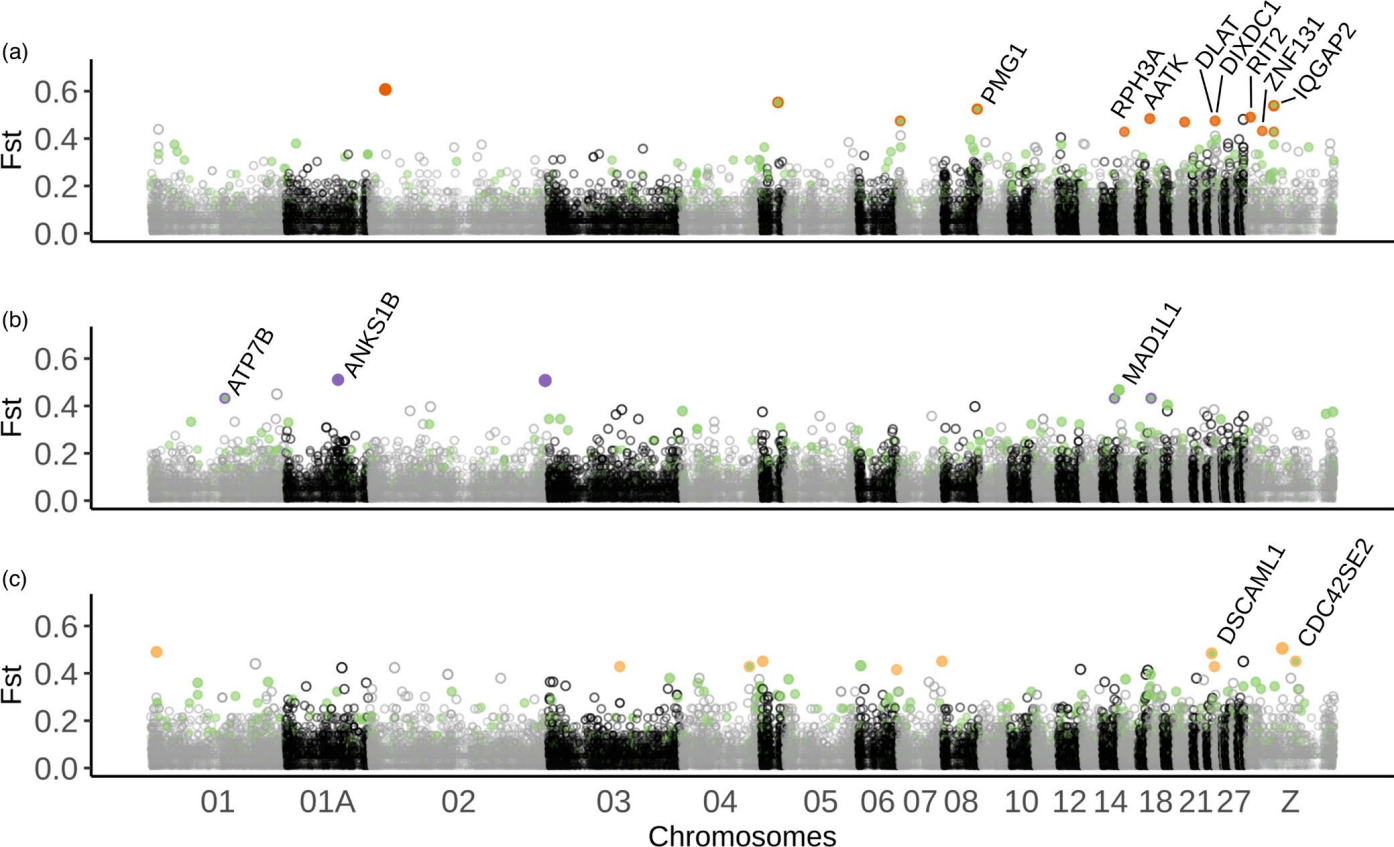
Manhattan plot of mean *F*_ST_ between urban and forest populations along the Great tit genome for (a) Barcelona, (b) Montpellier and (c) Warsaw. Dark orange (a), purple (b) and light orange points (c) represent significant outlier SNPs identified by the *F*_ST_‐outlier test Bayescan for each population pair, given with their associated genes in 5 kb. Green points represent outliers found with the multivariate RDA approach. A few SNPs were identified by both the Bayescan and the RDA methods and signalled as a green point circled with the colour used for the considered pair

First, Bayescan identified 15 outliers for Barcelona, 11 for Montpellier and 10 for Warsaw, distributed across 15 chromosomes and associated with 13 genes in 5kb upstream or downstream regions (Figure 4, *q*‐value < 0.1, see Figure S1). None of these outliers was shared between the three population pairs, revealing no convergence between cities. Second, the multivariate approach based on an RDA revealed a list of 1163 loci with outlier loading score (Figure S2) suggesting a high number of loci putatively undergoing weaker selection. These 1163 loci were associated with 561 genes.

GO analyses revealed the existence of overrepresented ontologies (*p* < 0.05 and at least 3 genes per GO). Among the most promising GO terms we found functions related to the nervous system (GO:0048846, axon extension involved in axon guidance; GO:0035418 protein localization to synapse; GO:0021987 cerebral cortex development; GO:0007274, neuromuscular synaptic transmission), the blood system (GO:0045777 positive regulation of blood pressure; GO:0042311, vasodilatation), hormonal response (GO:0071277, cellular response to oestrogen stimuli) and stress response (GO:0033555, multicellular response to stress), revealing functions potentially involved in adaptation to urban habitats. Detailed GO results are presented in Table S9 and Figure S3.

### 3.3 Evidence for mostly nonparallel differentially methylated regions between urban and forest environments

**FIGURE 5:**
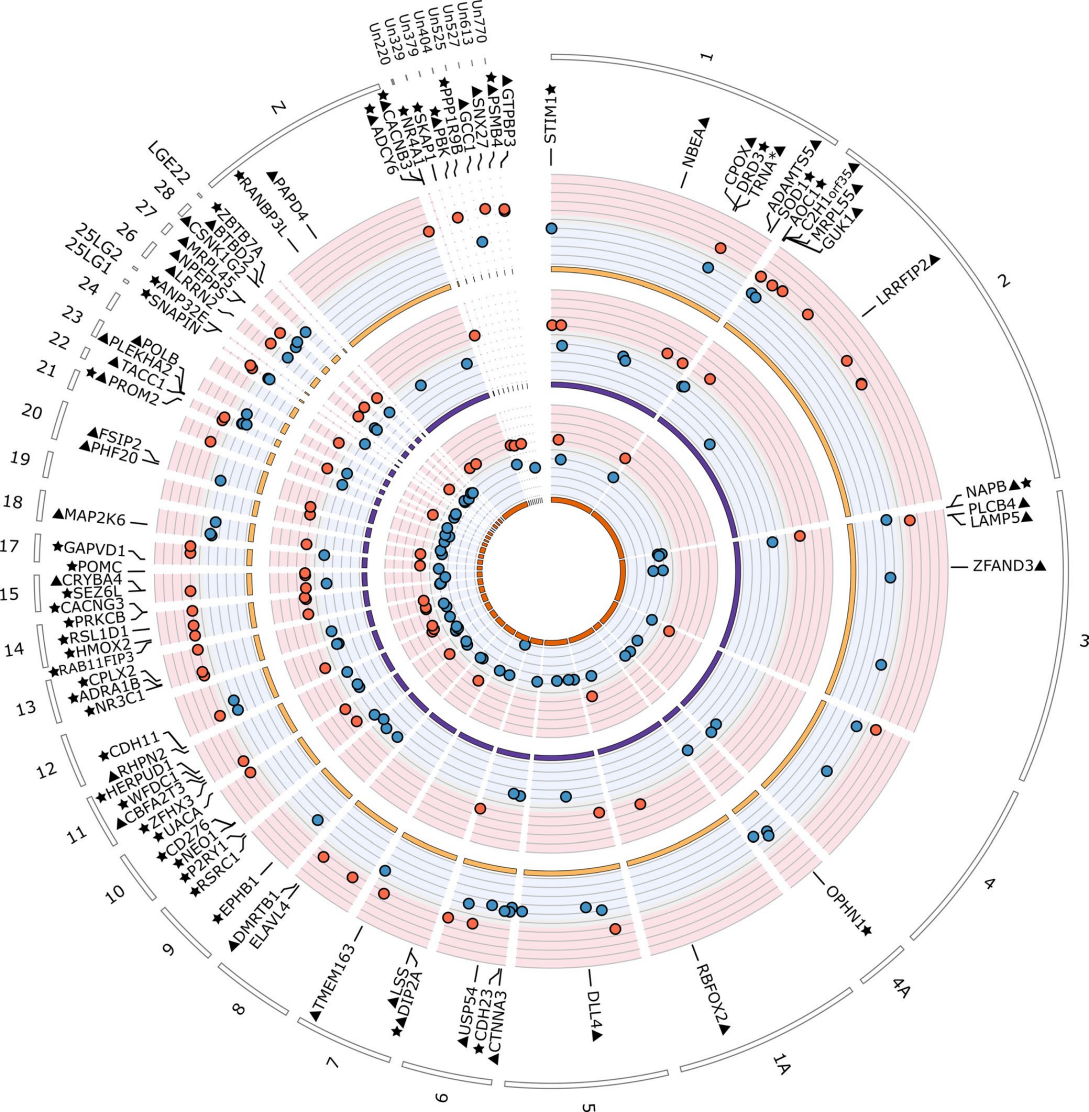
Circos plot of differentially methylated regions (DMRs) identified between populations of forest and urban great tits in and near Barcelona, Montpellier and Warsaw (from inner to outer circles). Red points show hypermethylated regions in urban great tits relatively to forest birds, and blue points show hypomethylated regions. For graphical clarity, only a subset of genes are represented: genes associated with the 10% most extreme DMR (triangles) and genes found associated with DMR in at least two cities (stars). Names of the genes found within 5 kb of the represented DMRs are given

We identified a total of 224 distinct DMRs between urban and forest great tits: 80 for Barcelona, 68 for Montpellier and 93 for Warsaw. Only 14 DMRs (6.25%) were found repeatedly in at least two comparisons, and only 3 were common to the three cities. 7 of these 14 parallel DMRs were in the same direction of methylation in urban compared to forest areas (Figure 5, Figure S4a). Barcelona urban birds presented significantly more hypomethylated than hypermethylated DMRs compared to Barcelona forest birds (χ^2^ = 11.25, *p* < 0.001), while no difference was found for Montpellier (χ^2^ = 0.941, *p* = 0.332) or for Warsaw (χ^2^ = 0.011, *p* = 0.917). DMRs were distributed across all the 32 chromosomes as well as on 37 unplaced scaffolds. 203 of the 224 different DMRs (91%) overlapped genes or 5 kb flanking regions. Among these 203 DMRs, 126 (62%) were directly located in gene bodies (*n* = 53, 26.1%), 48 (23.6%) in promotor (3 kb upstream to 300 bp downstream of genes, excluding TSS) or TSS sequences (300 bp upstream to 50 bp downstream of genes), and 26 (12.8%) in both gene body and promotor/TSS.

Following the procedure previously described, GO analyses on the pooled genes list revealed an overrepresentation of modules associated with the nervous system (GO:2000300, regulation of synaptic vesicle exocytosis; GO:0050804 modulation of synaptic transmission), immunity (GO:005728, negative regulation of inflammatory response; GO:0050852, T cell receptor signalling pathway), metabolic activity (GO:006816 calcium ion transport; GO:0055072, iron ion homeostasis, GO:0043087, regulation of GTPase activity), behaviour (GO:0007626, locomotory behaviour) and endocrine processes (GO:0044060: regulation of endocrine process). All enriched GO are presented in Table 2 and Figure S5.

**TABLE 2:** Significantly enriched GO associated with genes overlapping 5 kb windows around DMRs between forest and urban habitats

GO.ID	Term	Annotated	Significant	Expected	Weight	Barcelona	Montpellier	Warsaw
GO:0050804	Modulation of chemical synaptic transmission	190	10	2.92	0.016	CACNG3, CPLX2, OPHN1, PPP1R9B, PRKCB	CDH11	DRD3, EPHB1, NAPB, SNAPIN
GO:0042981	Regulation of apoptotic process	619	8	9.52	0.004	DIP2A, HERPUD1, NR4A1, SOD1,		ANP32E, RSL1D1, UACA
GO:0007626	Locomotory behavior	122	6	1.88	0.005	ADCY6, ELAVL4, SOD1		CDH23, DRD3, SEZ6L
GO:0006816	Calcium ion transport	173	6	2.66	0.024	CACNB3, CACNG3, PRKCB		CDH23, DRD3,STIM1
GO:0050728	Negative regulation of inflammatory response	61	5	0.94	0.011	PSMB4, SOD1,	CD276, WFDC1	PBK
GO:0046677	Response to antibiotic	91	5	1.4	0.012	ADCY6, SOD1	RSRC1	AOC1, DRD3
GO:0043087	Regulation of GTPase activity	237	5	3.65	0.025	GAPVD1, OPHN1, RANBP3L, SOD1	PROM2	PROM2
GO:2000300	Regulation of synaptic vesicle exocytosis	40	4	0.62	0.017	CPLX2	PRKCB	NAPB, SNAPIN
GO:0008217	Regulation of blood pressure	74	4	1.14	0.04	SOD1	POMC	ADRA1B, DRD3
GO:0044060	Regulation of endocrine process	22	3	0.34	0.012	NR3C1,	POMC	RAB11FIP3
GO:0055072	Iron ion homeostasis	42	3	0.65	0.014	NEO1, SOD1	HMOX2	
GO:0071559	Response to transforming growth factor beta	113	3	1.74	0.031	NR3C1, ZBTB7A, ZFHX3		
GO:0050852	T cell receptor signaling pathway	52	3	0.8	0.046	CACNB3, SKAP1	CD276	
GO:0007200	Phospholipase C‐activating G protein‐coupled receptor signaling pathway	40	3	0.62	0.048	ADRA1B	P2RY1	DRD3

**FIGURE 6:**
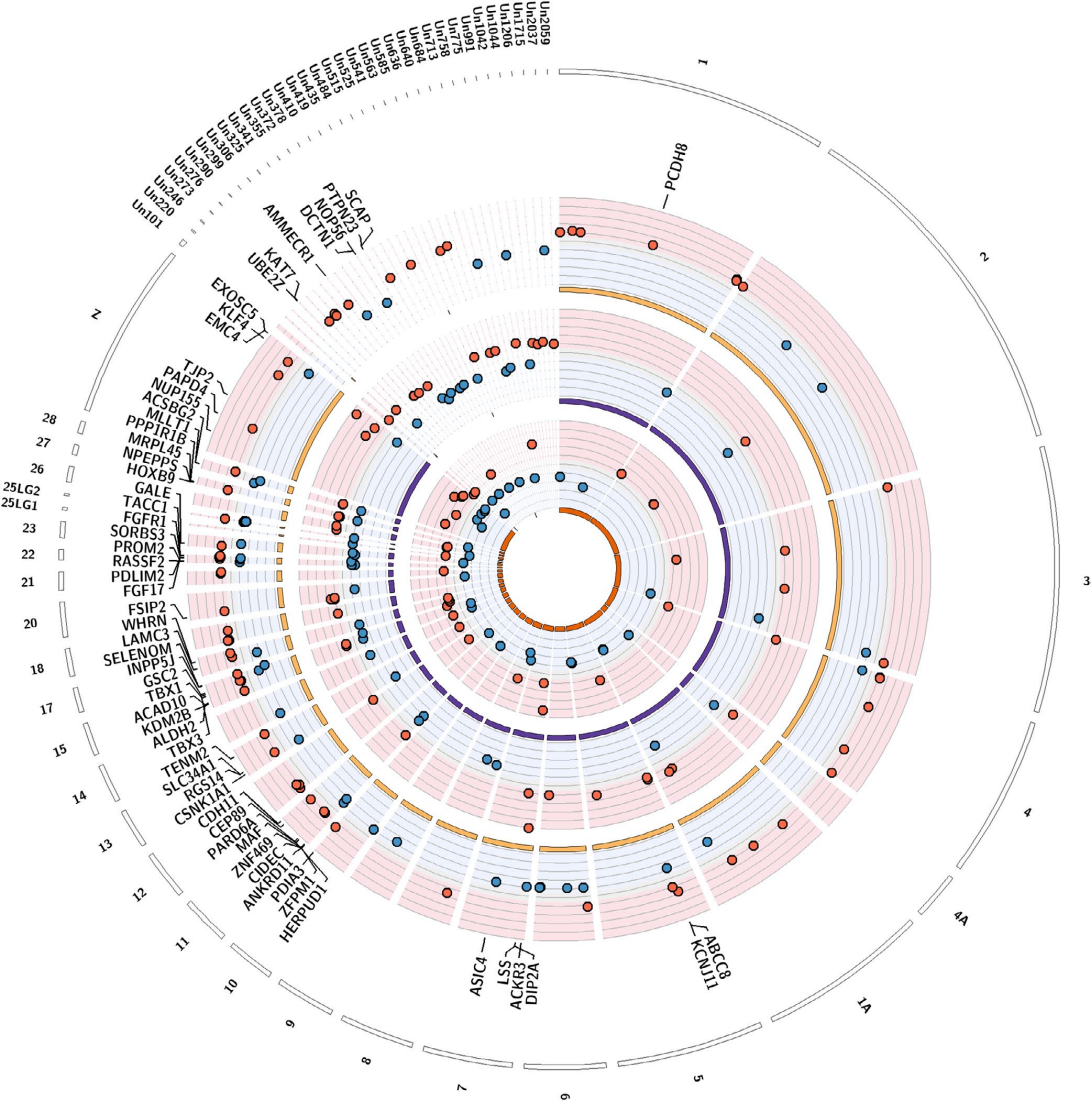
Circos plot of differentially methylated regions (DMRs) identified between females and males great tits in and near Barcelona, Montpellier and Warsaw (from inner to outer circles). Red points show hypermethylated regions in female great tits relatively to males, and blue points show hypomethylated regions. For graphical clarity, only a subset of genes are represented: genes associated with the 10% most extreme DMR. Names of a subset of the genes found within 5 kb of the represented DMRs are given

We also searched for DMRs between sexes, following the same procedure. We identified 206 DMRs associated with sex, of which 58 for Barcelona, 81 for Montpellier and 99 for Warsaw. Warsaw presented significantly more hyper than hypomethylated (in females compared to males) DMRs (χ^2^ = 5.878, *p *= 0.015), but it was not the case for Barcelona (χ^2^ = 0, *p *= 1) nor Montpellier (χ^2^ = 1.25, *p *= 0.264). DMRs were distributed on 29 chromosomes and 35 unplaced scaffolds. On a total of 206 DMRs, 181 (57.3%) were on genes or in a 5 kb upstream/downstream region around genes. GO analyses revealed enrichment of genes involved in development, growth and morphogenesis, among others (see detailed enriched GO Table 3, Figure 6 and Figure S6).

**TABLE 3:** Significantly enriched GO associated with genes overlapping 5 kb windows around DMRs between sexes

GO.ID	Term	Annotated	Significant	Expected	Weight	Barcelona	Montpellier	Warsaw
GO:1903706	Regulation of hemopoiesis	172	8	2.28	0.050	KAT7, RARA, RUNX1, TJP2	CARD11, KAT7, PTN, RASSF2	
GO:1902930	Regulation of alcohol biosynthetic process	20	3	0.27	0.039	SCAP	SCAP, SOD1, VDR	FGFR1
GO:1902652	Secondary alcohol metabolic process	53	4	0.7	0.013	LSS, SCAP	LSS, SCAP, SOD1	FGFR1
GO:0060173	Limb development	123	4	1.63	0.028	RARA	PTN, TBX3	FGFR1, TBX3
GO:0060117	Auditory receptor cell development	13	3	0.17	0.026	WHRN	SOD1	FGFR1
GO:0046887	Positive regulation of hormone secretion	46	3	0.61	0.016			BLK, FGFR1, NMB
GO:0046849	Bone remodeling	46	3	0.61	0.023	TPH1	PTN, RASSF2	
GO:0043087	Regulation of GTPase activity	237	6	3.15	0.006	MLST8	PROM2, SOD1	LRRK2, PROM2, RGS14, STARD8
GO:0042981	Regulation of apoptotic process	619	19	8.22	0.002	DIP2A, RARA, UBE2Z	BAG5, CARD11, CIDEC, DIP2A, GRK5, RASSF2, SOD1, TBX3	ANP32E, BLK, FGFR1, HERPUD1, KDM2B, LRRK2, PDIA3, PHLDA1, TBX3
GO:0035264	Multicellular organism growth	93	4	1.23	0.033	RARA, SELENOM	ANKRD11, SOD1	**ANKRD11**, SELENOM
GO:0032436	Positive regulation of proteasomal ubiquitin‐dependent protein catabolic process	46	4	0.61	0.018		CSNK1A1	CSNK1A1, HERPUD1, LRRK2, STUB1
GO:0032091	Negative regulation of protein binding	50	3	0.66	0.028		ZFPM1	LRRK2, STUB1
GO:0031647	Regulation of protein stability	152	5	2.02	0.048	LSS	BAG5, LSS, RASSF2	LRRK2,STUB1
GO:0009790	Embryo development	625	15	8.3	0.049	MDFI, PCDH8, RARA, WHRN	ANKRD11, HOXB9, PTN, SOD1,TBX1,TBX3, ZFPM1	**ANKRD11**, ASCL2, DNMT3A, FGFR1, KDM2B, PCDH8, TBX3
GO:0009267	Cellular response to starvation	85	4	1.13	0.044	**PIK3C2B**	XPR1	LRRK2,WDR24
GO:0007218	Neuropeptide signaling pathway	46	3	0.61	0.023			GALR2, NMB, **POMC**
GO:0003151	Outflow tract morphogenesis	46	3	0.61	0.046	RARA	TBX3, ZFPM1	TBX3
GO:0001666	Response to hypoxia	91	4	1.21	0.023	NPEPPS, SCAP	NPEPPS, SCAP	ASCL2, LIMD1
GO:0000122	Negative regulation of transcription by RNA polymerase II	454	12	6.03	0.017	RARA	CBFA2T2, **SCAF4**, TBX1, TBX3, VDR, ZFPM1	CSNK1A1, HERPUD1, LRRK2, STUB1

Note

Bold genes represent when a DMR overlapped a gene boy and underlined genes, when DMR overlapped TSS or promotor regions.

Almost twice more sex DMRs were shared between locations (11.7%) than between habitats (6.25%, see Figure S4a,b; *z*‐test: χ^2^‐squared = 3.885, *p* = 0.049). When taking into account the direction of methylation difference, 7 sex DMRs were shared between at least two cities (9.7%) which was three times more than for habitat DMRs (3.1%; *z*‐test: X‐squared = 7.904, *p* = 0.005).

Finally, we also explored patterns of methylation associated with candidate genes linked to behavioural, morphological and phenological traits that are known to differ between forest and city great tits, and which were covered by our sequencing. While none of these genes shows strong methylation difference, we provide informative plots that could be used by other studies that wish to focus on these genes and provide informative plots that could be used by other studies that wish to focus on these genes (see Appendix 1 and Figures S7–S11 for CLOCK, COL4A5, DRD4, EGR1 and FOXP2).

## 4 DISCUSSION

The urban sprawl is a worldwide phenomenon deeply affecting the environment and thus requiring fast adaptive responses in city dwellers. While a large body of literature already describes a myriad of phenotypic shifts in urban populations of numerous species (Chamberlain et al., 2009; Lowry et al., 2013; Sepp et al., 2018), the molecular bases of these shifts and their evolutionary implications remain yet to be documented and understood. This study uses genomic and epigenomic analyses to decipher the potential molecular bases implicated in phenotypic shifts and adaptation in several urban populations of a passerine bird, the Great tit. Note that this species shows largely parallel phenotypic shifts across its range in terms of morphology and life history (Biard et al., 2017; Thompson et al., 2021). In our study, genomic analyses revealed weak yet significant average differentiation between urban and forest populations, suggesting ongoing gene flow and limited drift in urban populations. These analyses also identified a limited number of loci putatively under strong selection, nonrepeated between pairs, and numerous loci supposedly under weaker selection, compatible with a polygenic model of evolution. On the epigenomic side, while we found weak average differentiation of the methylome between urban and forest birds, suggesting an absence of genome‐wide epigenetic deregulations, we identified several differentially methylated regions between urban and forest birds, mostly nonrepeated between pairs. Genes associated with either genomic footprints of divergent selection or differentially methylated regions had relatively similar functions, related to the nervous system, metabolism, immunity and behaviour, that have been repeatedly convicted in other studies (Riyahi et al., 2017). Hence, by identifying nonrepeated genetic and epigenetic responses among replicated forest‐urban population pairs, our findings support the hypothesis of mostly nonparallel rapid de novo adaptation to similar environments via both genetic and epigenetic mechanisms. Our results are in line with accumulating evidence that polygenic adaptation and epigenetic reprogramming may be involved in quick phenotypic shifts in response to rapidly emerging constraints such as urbanization.

Overall genetic differentiation between populations was relatively low (*F*_ST_ ranging from 0.009 to 0.034), although higher than what has been found at a much larger scale across the species distribution (e.g. *F*_ST_ around 0.01 between the UK and Spanish or between French and Spanish populations, Laine et al., 2016). Low but significant differentiation levels are in line with previously documented genetic divergences between city and forest great tit populations (Perrier et al., 2018; Senar & Björklund, 2020), and altogether suggests important gene flow, large effective population sizes and limited genetic drift at multiple spatial scales (Kvist et al., 2003). This overall genetic context is particularly suitable to search for genomic footprints of divergent selection between urban and forest populations, which would easily be identifiable above the neutral level of genetic differentiation.

We found a limited number of strong footprints of divergent selection, which is in line with previous results in Montpellier (Perrier et al., 2018), and with the broader work of Salmón et al. (2021) across nine European cities. Similarly to low levels of parallelism in allele frequency changes between cities observed by previous studies (Reid et al., 2016; Salmón et al., 2021), and despite similarities in phenotypic shifts, none of these outliers were shared between cities. This result suggests limited parallel evolution, supporting a scenario of independent de novo evolution between cities and/or different selection pressures between cities. Indeed, there may be multiple evolutionary solutions to the same environmental challenges (Losos, 2011) and multiple traits are linked to the same functional outcome (Thompson et al., 2017). Besides, the identification of numerous outliers by the multivariate framework applied at the scale of all six sampling sites supports a model of polygenic urban adaptation implicating multiple genes, biological pathways and phenotypic traits (Boyle et al., 2017). Polygenic adaptation is a reasonable expectation in urban habitats since the multiple new environmental conditions in cities most probably result in many novel selective pressures acting on a multitude of functional traits (Shochat et al., 2006), and because many of these traits may be quantitative, and genetically correlated (Lande, 1979). Further polygenic analyses on more samples and more markers (i.e. whole genome data) are however required in order to estimate the potential effect of each genetic variant implicated in the adaptation to life in the city (Robinson et al., 2014; Zhou & Stephens, 2012).

Several genomic footprints of divergent selection between urban and forest environments were in, or in the vicinity of, genes that have already been described as playing a role in neuronal development, behaviour or cognitive abilities. In particular, the NR4A2 gene plays an important role in recognition of novel objects and memory in mice (McNulty et al., 2012). Reaction to novel objects and novel food is known as one of the main factors determining the capacity of a species to thrive in an urban environment (Lowry et al., 2013). The DCX gene is related to neuronal plasticity (Kim et al., 2006) and experimental approaches revealed that artificial light at night induces an overexpression of this gene linked to a change in behaviour and expression of depressive‐like behaviour in crows (Taufique et al., 2018). Finally, the CHRNA1 gene is associated with aggressive behaviour in chicken (Buitenhuis et al., 2009), and higher aggressiveness is commonly observed and hypothesized as adaptive in urban bird populations (Sprau & Dingemanse, 2017). Besides, the gene ontology enrichment analysis, performed on the entire set of genes identified via the outlier genome scan, reinforced these findings since multiple enriched GO terms were associated with the nervous system and stress response as well as hormonal response (Table 2). These results are informative on the type of traits involved in avian urban adaptation in cities and corroborate previous results from (Salmón et al., 2021; Sih & Del Giudice, 2012; Sol et al., 2013) suggesting that natural selection repeatedly acted on neuronal, behavioural and cognitive traits that could contribute to the phenotypic shifts described in urban great tits (i.e. more aggressive and exploratory birds, with higher breath rate; A. E. Caizergues, A. Grégoire, R. Choquet, S. Perret, & A. Charmantier, unpublished data; Senar et al., 2017; Torné‐Noguera et al., 2014).

Contrary to the common prediction that living in cities is likely to influence epigenomes (McNew et al., 2017; Watson et al., 2021), no genome‐wide pattern of differentiation in methylation between urban and forest great tits was detected. However, we observed a difference in mean methylation level between birds from Warsaw and Barcelona on their autosomes, as well as between males and females on the Z chromosome, showing that methylation differences were identifiable. In addition, we found no mean difference in methylation level between habitats, revealing that urbanization did not strongly affect overall methylation levels in a specific direction. This overall low differentiated methylation context is perfectly suitable to investigate more localized zones that could differ in their levels of methylation. Note that the strong methylation contrast between males and females on sex chromosomes (Figure 2) is in line with previous reports in vertebrates (Teranishi et al., 2001; Waters et al., 2018) showing that methylation plays a major role in sex differentiation via regulation of gene expression and genetic imprinting.

Despite the nonsignificant effect of habitat on overall methylation levels, we found a large number of DMRs between pairs of forest and urban populations, suggesting that urbanization did affect particular regions of the genome. DMR were less likely to occur within a gene body than by chance, but it was not the case for promotor or TSS regions. This latter result contrasts with Watson and collaborators (Watson et al., 2021) who recently found an under‐representation of DMS in both gene body and regulatory regions in urban great tits from Malmö (Sweden). Across the three cities, 62% of DMR were directly localized in gene bodies and/or TSS or promotor regions, suggesting a potential role in gene expression modulation. Direction of methylation patterns did not follow any consistent pattern (no over‐representation of hypo‐ or hypermethylated DMR in urban birds, Figure 5), in line with Watson and collaborators’ analyses on blood sample. However, birds in Barcelona presented significantly more hypomethylated DMR than hypermethylated ones.

Only a limited number of urbanization‐linked DMR were shared between two or more locations (note that the particular sampling pattern in Barcelona (see methods) could potentially affect the results found for this city). In contrast, three times more sex‐linked DMRs were found in two locations or more. This comparison suggests that urbanization‐linked epigenetic modifications most probably do not occur in a parallel way across cities, but rather that each city might have its particular epigenetic response. Indeed, in the emerging field of urban evolutionary biology, cities are often regarded as valuable replicates of human‐altered habitats (Donihue & Lambert, 2015; Santangelo et al., 2020), and it is often expected that parallel adaptive responses to similar selective pressures will occur. This expectation is particularly strong when phenotypes show parallel changes, as is the case for the Great tit, which is consistently smaller and lays earlier and smaller broods in the various cities where it has been studied, compared to forest habitats (Caizergues et al., 2018; Seress et al., 2020). However, as discussed above, parallel adaptation to similar environmental conditions should not be expected in the case of independent evolution, especially for multilocus traits. Additionally, cities are different from each other because of a wide array of climatic, cultural, historical and socioeconomic factors (Szulkin, Garroway, et al., 2020; Szulkin, Munshi‐South, et al., 2020). In fact, besides the obvious differences in cities’ climatic conditions depending on their position on the globe, land use, fragmentation and pollutants levels can also largely vary across cities (Cárdenas Rodríguez et al., 2016). In a general way, pollutants are known to affect DNA methylation and result in both hypo‐ and hypermethylation, but the patterns of change observed largely rely on the pollutant involved (Head, 2014). Hence differences in cohorts of pollutants present in cities could be responsible for differences in patterns of methylations. Taken together, the results of the present study highlight the importance of questioning the assumption that cities are replicated environments that can be considered similar, and parallel evolution across cities may be the exception rather than the norm.

As mentioned earlier, increasing evidence suggests that DNA methylation can be associated with environmental and stress factors (env: Foust et al., 2016, stress: Sun et al., 2018) especially during early development (Meaney & Szyf, 2005). Here, we found four genes (POMC, ADAMTS3, PAPD4 et GCC1), associated with DMR that were previously described in great tits as undergoing major changes in methylation levels in case of exposure to higher levels of pollutants (Mäkinen et al., 2019). Notably, the functions of these genes remain to be determined, and they could thus be interesting to target in future studies. In addition, the past literature has repeatedly found SERT and DRD4 as two major genes involved in urban‐specific avian human avoidance (or wariness) behaviours (see for example, in the Great tit (Riyahi et al., 2015), in the blackbird (Garroway & Sheldon, 2013), in the black swan (Van Dongen et al., 2015) and in the burrowing owl (Mueller et al., 2020)). In this study, while urban great tits show higher levels of aggressiveness in at least two of the cities (A. E. Caizergues, A. Grégoire, R. Choquet, S. Perret, & A. Charmantier, unpublished data; Riyahi et al., 2017) we found no DMR associated with these two genes in either of the three forest‐city pairs. However, we found a significant urban‐related change in methylation linked to the DRD3 gene, belonging to the same gene family as DRD4 and known to be similarly involved in chicken aggressive behaviour (Li et al., 2016). In line with these results, GO analyses revealed enrichment in genes associated with neuronal functions, behaviour, but also blood, immune and endocrine systems (Table 2, Figure S5), revealing the potential need of physiological adjustments in urban habitats. Surprisingly, a recent study on great tit differences of methylation between city and forest habitats in another European city found no GO enrichment in blood, while some in liver tissue (Watson et al., 2021; note that they investigated DMSs, Differentially Methylated Sites, which differs from DMRs identified here and do not provide the same information). These contrasted results highlight the fact that methylation patterns highly depend on the analysed tissues (Derks et al., 2016), and show, once more, that urban linked methylation might not be similar from one city to another. As reviewed by Husby (2020), the use of blood sample in methylation studies comes with several potential limitations. For instance, as previously mentioned (1) blood methylation patterns might not reflect the ones in other tissues, (2) methylation shifts are known to change gene expression in the tissue sampled, but little is known on their effects on other tissues and (3) DNA methylation patterns can undergo seasonal variation (Viitaniemi et al., 2019) which is especially true for blood because of rapid cellular turnover. Hence, analyses on multiple tissues and life‐stages replicated in multiple pairs of urban and forest populations are required, to draw a broader view of the impact of urbanization on global methylation patterns and to understand replicated parallel occurrence across cities. However, tissue‐specific and age‐specific analyses in multiple individuals across several pairs of urban and forest environments pose major technical, budget and ethical limitations and should be coordinated very carefully. Additionally, specific drivers of shifts in methylation remain to be disentangled to understand which environmental factors are responsible for which change in methylation. To do so, experimental settings manipulating environmental factors such as performed by Mäkinen et al. (2019) would be particularly useful. More integratively, information on how shifts in methylation patterns affect phenotype, fitness and adaptation often remain elusive (Sepers et al., 2019). To our knowledge, a limited number of studies attempted to link methylation and expression levels in natural population contexts (Derks et al., 2016; Laine et al., 2016), and even fewer in urban habitats (but see e.g. McNew et al., 2017; Watson et al., 2021). Hence, future work might need to tackle the question of the origin and adaptive significance of these variations in a controlled framework.

Finally, the consideration of sample size in terms of number of individuals sampled per population as well as number of replicated populations is undoubtfully of a major importance. Although this study is in the upper range of sample sizes found in previous studies on parallelism, we may still have limited power to detect parallel marks of evolution if methylation shifts at these marks are relatively low. To date, no power analysis has been developed specifically for (epi)genomic parallelism and such methodological development could be extremely useful to evaluate the confidence in our studies. Future studies should aim at increasing individual sample size per population and conducting broader geographic scale analyses with more replicated population pairs (see e.g. Salmón et al. (2021) across nine European cities) to increase the power to detect parallelism in epigenomic responses to urbanization.

This study identified both genomic footprints of selection and differentially methylated regions between urban and forest populations, suggesting that both genetic and epigenetic processes could play a role in rapid adaptation to urban habitat. To our knowledge, our study is the first to use replicated pairs of city and forest populations to study modifications of methylation in urban habitats. This study design revealed limited evidence for parallelism between cities both at the genetic and epigenetic levels, suggesting that cities might not present exactly similar environmental conditions or that different genetic and epigenetic pathways are involved in adaptation to urban environmental conditions, although possibly associated with similar biological functions. This study finally highlights the need to unravel both environmental origins and evolutionary implications of methylation shifts, in order to understand to which extent environmentally induced methylation can contribute to adaptation.

## CONFLICT OF INTEREST

We declare we have no competing interests.

## Supporting information

Supplementary Material

## Data Availability

DNA sequences: RAD‐seq GenBank accessions SRR17125662‐SRR17125721, RRBS‐seq GenBank accessions SRR17145241‐SRR17145300; NCBI project PRJNA786007. Scripts: Github repository https://github.com/AudeCaizergues/Epigenetics_and_the_city
