# Supplementary material for: Epigenetics and the city: Non‐parallel DNA methylation modifications across pairs of urban‐forest Great tit populations
Source: Evol Appl. 2022 Jan 4;15(1):149–65. doi: 10.1111/eva.13334 (PMC8792475; doi:10.1111/eva.13334)
Supplement: Supplementary file 1 — Supplementary Material [file EVA-15-149-s001.pdf]

SUPPLEMENTARY INFORMATION

APPENDIX

Appendix 1: Investigating methylation shifts on candidate genes

Previous studies on birds (particularly great tits) in urban or non-urban context highlighted several genes involved in phenotypic shifts associated with urbanization:

- DRD4 and SERT: involved in novelty seeking, risk taking and exploratory behaviour (Mueller, Partecke, Hatchwell, Gaston, & Evans, 2013; Riyahi, Sánchez-Delgado, Calafell, Monk, & Senar, 2015)
- CLOCK: involved in circadian behaviour (e.g. Steinmeyer, Mueller, & Kempenaers, 2009)
- COL4A5: involved in bill morphology (Bosse et al., 2017)
- EGR1: involved in learning and memory (Laine et al., 2016)
- FOXP2: involved in song learning (Laine et al., 2016)
- GP2: involved in modulating immune response (Lindner et al., 2021)
- NR5A1: involved in regulating female reproduction, in particular ovarian functioning (Lindner et al., 2021)

We explored patterns of methylation associated with these gene and provide methylation plots that could be used in future studies focusing on these genes. Note that among this list of genes, only DRD4, CLOCK, COL4A5, EGRI and FOXP2 were sequenced with enough quality in our dataset and were hence plotted in Figure S7 to S11. We do not detect any clear pattern of methylation shifts between urban and forest birds on any of these genes. These results suggest that if a difference in expression of these genes is responsible for the phenotypic shifts observed in the urban habitat, such difference in expression is probably not due to a difference in methylation in blood tissues.

SUPPLEMENTARY TABLES

Table S1: RRBS reads filtering and mapping success reports per sample.

Sample	Raw PE reads (million)	Filtered PE reads (million)	Unique mapping efficiency
bar1	16.40	15.91	45.20%
bar2	20.58	20.25	43.10%
bar3	22.49	22.13	45.20%
bar4	18.43	18.10	41.10%
bar5	22.68	22.02	43.80%
bar6	19.63	19.29	43.10%
bar7	20.35	20.06	44.20%
bar8	27.13	26.67	44.00%
bar9	19.39	18.96	44.30%
bar10	18.70	18.40	41.40%
bar11	20.68	20.26	43.10%
bar12	20.48	20.18	42.20%
bar13	20.20	19.75	43.50%
bar14	19.88	19.54	44.40%
bar15	13.60	13.42	43.50%
bar16	18.41	18.05	45.40%
bar17	16.31	16.08	40.10%
bar18	17.80	17.51	41.00%
bar19	20.11	19.76	43.90%
bar20	19.39	19.03	43.40%
mtp1	18.72	18.43	35.90%
mtp2	20.70	20.31	43.20%
mtp3	22.82	22.24	41.30%
mtp4	27.86	27.11	45.10%
mtp5	25.34	24.96	43.60%
mtp6	17.58	17.37	38.40%
mtp7	21.25	20.67	43.80%
mtp8	19.51	19.24	38.40%
mtp9	23.91	23.24	39.70%
mtp10	20.64	20.35	44.50%
mtp16	23.45	22.91	41.70%
mtp17	19.02	18.72	41.70%
mtp18	15.59	15.36	37.90%
mtp19	15.85	15.64	40.60%
mtp20	16.55	16.19	42.10%
mtp21	22.14	21.82	41.50%
mtp22	20.23	19.82	41.70%
mtp23	16.51	15.95	43.70%
mtp24	17.71	17.30	41.70%
mtp25	19.27	18.88	40.30%
var1	17.92	17.66	43.50%
var2	19.71	19.45	43.40%
var3	17.52	17.17	43.50%
var4	17.09	16.71	44.70%
var5	14.76	14.53	44.10%
var6	18.46	18.13	42.30%
var7	21.47	21.13	46.30%
var8	19.78	19.10	45.30%
var9	15.82	15.36	44.30%
var10	22.33	21.95	44.80%
var11	16.23	15.97	44.00%
var12	16.67	16.41	42.20%
var13	19.54	19.15	44.90%
var14	24.50	24.03	47.10%
var15	16.50	16.13	46.20%
var16	15.77	15.39	45.20%
var17	19.56	19.14	44.20%
var18	20.34	19.86	45.50%
var19	14.65	14.41	42.90%
var20	16.48	16.18	42.40%

Table S2: Redundancy analysis (RDA) performed on the genetic data including the Z chromosome.

	adjusted R-squared	P-value	RDA1	RDA2	RDA3	RDA4
Full RDA	0.018	0.001	Variance explained by axes			
			0.024	0.021	0.021	0.019
Variables			Biplot scores			
City – Montpellier		0.001	0.570	-0.819	0.059	-0.039
City – Warsaw			0.381	0.883	0.214	0.170
Habitat – Urban		0.001	-0.210	-0.100	0.968	-0.089
Sex – Male		0.004	0.184	0.143	-0.036	-0.972
Partial RDA for city	0.012	0.001	Variance explained by axes			
			0.025		0.022	
Variable			Biplot scores			
City – Montpellier		0.001	0.630		-0.776	
City – Warsaw			0.356		0.934	
Partial RDA for habitat	0.004	0.001	Variance explained by axis			
			0.022			
Variable			Biplot score			
Habitat – Urban		0.001	0.999			
Partial RDA for sex	0.002	0.004	Variance explained by axis			
			0.020			
Variable			Biplot score			
Sex – Male		0.005	-0.999			

Table S3: Redundancy analysis (RDA) performed on the genetic data without Z chromosome.

	adjusted R-squared	P-value	RDA1	RDA2	RDA3	RDA4
Full RDA	0.016	0.001	Variance explained by axes			
			0.024	0.021	0.021	0.017
Variables			Biplot scores			
City – Montpellier		0.001	0.573	-0.818	0.049	-0.024
City – Warsaw			0.382	0.889	0.227	0.110
Habitat – Urban		0.001	-0.215	-0.119	0.967	-0.073
Sex – Male		0.26	0.140	0.096	-0.033	-0.985
Partial RDA for city	0.012	0.001	Variance explained by axes			
			0.025		0.022	
Variable			Biplot scores			
City – Montpellier		0.001	0.624		-0.781	
City – Warsaw			0.362		0.932	
Partial RDA for habitat	0.004	0.001	Variance explained by axis			
			0.022			
Variable			Biplot score			
Habitat – Urban		0.001	0.999			
Partial RDA for sex	0.0004	0.373	Variance explained by axis			
			0.018			
Variable			Biplot score			
– Urban		0.369	-0.999			

Table S4: Fst estimation averaged on autosomes between pairs of subpopulations. 95% confidence computed intervals in brackets were computed using StAMPP package with 1000 bootstrap.

	Mont-Rur	Mont-Urb	Var-Rur	Var-Urb	Bar-Rur
Mont-Urb	0.017 (0.017 – 0.017)	-	-	-	-
Var-Rur	0.018 (0.018 – 0.018)	0.015 (0.014 – 0.015)	-	-	-
Var-Urb	0.014 (0.014 – 0.015)	0.011 (0.011 – 0.011)	0.015 (0.014 – 0.015)	-	-
Bar-Rur	0.018 (0.018 – 0.018)	0.015 (0.014 – 0.015)	0.018 (0.018 – 0.018)	0.014 (0.014 – 0.014)	-
Bar-Urb	0.011 (0.011 – 0.011)	0.008 (0.007 – 0.008)	0.011 (0.011 – 0.011)	0.008 (0.007– 0.008)	0.014 (0.014 – 0.014)

Table S4: Fst estimation averaged on Z chromosome between pairs of subpopulations. 95% confidence computed intervals in parenthesis were computed using StAMPP package with 1000 bootstrap.

	Mont-Rur	Mont-Urb	Var-Rur	Var-Urb	Bar-Rur
Mont-Urb	0.013 (0.006 – 0.018)	-	-	-	-
Var-Rur	0.016 (0.009 – 0.021)	0.020 (0.013 – 0.025)	-	-	-
Var-Urb	0.025 (0.019 – 0.031)	0.025 (0.020 – 0.032)	0.020 (0.015 – 0.025)	-	-
Bar-Rur	0.015 (0.010 – 0.019)	0.017 (0.011 – 0.022)	0.014 (0.009 – 0.019)	0.018 (0.015 – 0.024)	-
Bar-Urb	0.031 (0.026 – 0.036)	0.041 (0.032 – 0.047)	0.028 (0.022 – 0.034)	0.036 (0.030– 0.043)	0.014 (0.009 – 0.021)

Table S6: Redundancy analysis (RDA) performed on the methylation data including the Z chromosome.

	adjusted R-squared	P-value	RDA1	RDA2	RDA3	RDA4
Full RDA	0.007	0.001	Variance explained by axes			
			0.021	0.019	0.018	0.016
Variables			Biplot scores			
City – Montpellier		0.001	0.210	0.259	-0.648	0.685
City – Warsaw			-0.603	-0.770	0.198	-0.067
Habitat – Urban		0.03	-0.256	0.286	0.669	0.637
Sex – Male		0.001	0.736	-0.562	0.263	0.269
Partial RDA for city	0.003	0.001	Variance explained by axes			
			0.021		0.018	
Variable			Biplot scores			
City – Montpellier		0.001	-0.356		0.934	
City – Warsaw			0.986		-0.161	
Partial RDA for habitat	0.001	0.300	Variance explained by axis			
			0.019			
Variable			Biplot score			
Habitat – Urban		0.181	0.999			
Partial RDA for sex	0.003	0.001	Variance explained by axis			
			0.021			
Variable			Biplot score			
Sex – Male		0.001	0.999			

Table S7: Redundancy analysis (RDA) performed on the methylation data without Z chromosome.

	adjusted R-squared	P-value	RDA1	RDA2	RDA3	RDA4
Full RDA	0.006	0.001	Variance explained by axes			
			0.021	0.019	0.018	0.016
Variables			Biplot scores			
City – Montpellier		0.001	0.231	-0.204	-0.666	0.679
City – Warsaw			-0.645	0.761	0.247	-0.058
Habitat – Urban		0.019	-0.261	-0.365	0.632	0.632
Sex – Male		0.001	0.698	0.569	0.326	0.289
Partial RDA for city	0.003	0.001	Variance explained by axes			
			0.020		0.018	
Variable			Biplot scores			
City – Montpellier		0.001	-0.357		0.933	
City – Warsaw			0.987		-0.159	
Partial RDA for habitat	0.001	0.176	Variance explained by axis			
			0.019			
Variable			Biplot score			
Habitat – Urban		0.168	0.999			
Partial RDA for sex	0.003	0.001	Variance explained by axis			
			0.021			
Variable			Biplot score			
Sex – Male		0.001	0.999			

Table S8: Results of Type I ANOVA performed on mean methylation level of cytosines in a CpG context per individual, including: habitat (rural vs urban), city (Barcelona, Montpellier and Warsaw) and sex as explanatory variables. The first part of the table shows results of the analysis on autosomes and the second part shows results of the analysis on the Z chromosome only.

Autosomes					
Effect	Df	Sum Sq	Mean Sq	F value	P
Habitat	1	0.416	0.416	0.511	0.478
Sex	1	1.041	1.041	1.278	0.263
City	2	5.408	2.704	3.319	0.044
Habitat* City	2	0.316	0.158	0.194	0.825
Residuals	53	42.174	0.8146		
Z chromosome					
Effect	Df	Sum Sq	Mean Sq	F value	P
Habitat	1	0.012	0.012	0.011	0.915
Sex	1	133.584	133.584	125.077	1.45x10-15
City	2	0.979	0.489	0.458	0.635
Habitat* City	2	1.1879	0.594	0.556	0.577
Residuals	53	56.605	1.068		

Table S9: Significantly enriched GO associated with genes overlapping 5kb windows around genomic outliers between forest and urban habitats.

GO.ID	Term	Annotated	Significant	Expected	Weight	Associated Genes
GO:2000618	regulation of histone H4-K16 acetylation	5	3	0.34	0.003	ATG5, AUTS2, SIRT1
GO:1902287	semaphorin-plexin signaling pathway invo...	7	3	0.48	0.009	PLXNA1, PLXNA2, PLXNA4
GO:1901842	negative regulation of high voltage-gate...	3	3	0.21	0.000	DYSF, GEM, RRAD
GO:0090335	regulation of brown fat cell differentia...	10	4	0.69	0.005	FTO, PIM1, PRDM16, SIRT1
GO:0071391	cellular response to estrogen stimulus	6	3	0.41	0.006	AR, CRHBP, ESR1
GO:0071277	cellular response to calcium ion	28	7	1.93	0.002	CRHBP, ECT2, HPCA, RASAL1, SYT1, SYT11, SYT9
GO:0050953	sensory perception of light stimulus	57	6	3.92	0.026	CDH23, CRYBB2, NYX, PCDH15, RPE65, WHRN
GO:0050765	negative regulation of phagocytosis	7	3	0.48	0.009	ATG5, DYSF, SYT11
GO:0048846	axon extension involved in axon guidance	23	4	1.58	0.026	NRP2, PLXNA4, SEMA4G, SLIT3
GO:0048026	positive regulation of mRNA splicing vi…	6	3	0.41	0.006	HMX2, NCBP1, NUP98
GO:0045777	positive regulation of blood pressure	9	3	0.62	0.026	ADRA1D, GRIP2, WNK1
GO:0044351	macropinocytosis	6	3	0.41	0.006	DOCK2, MAPKAPK2, SNX33
GO:0043410	positive regulation of MAPK cascade	197	15	13.56	0.013	ADRA1D, ADRB2, APP, AR,AXIN1, EPHA8, FGFR3, MTURN, NRG1, PDGFRB, PLCB1, RET, ROR1, ROR2, SH3RF2
GO:0043087	regulation of GTPase activity	194	17	13.36	0.025	AGAP3, ARHGAP17, ARHGAP45, ARHGEF12, DOCK2, ECT2, IQGAP2, MLST8, PLCB1, PLXNA1, PLXNA2, PLXNA4, RASAL1, RDX, RGS8, SIPA1L1, WNK1
GO:0042535	positive regulation of tumor necrosis fa...	6	3	0.41	0.006	APP, HSPB1, MAPKAPK2
GO:0042311	vasodilation	13	4	0.89	0.010	ADRB2, ATG5, CPS1, NOS1
GO:0035418	protein localization to synapse	26	5	1.79	0.009	BSN, GPC4, GRIP2, HSPB1, NRXN1
GO:0033555	multicellular organismal response to str...	37	6	2.55	0.006	BRINP1, DBH, NOS1, NR4A2, PPP3CA, RET
GO:0021987	cerebral cortex development	56	8	3.86	0.013	CCDC85C, CNTNAP2, DCX, HIF1A, NRP2, PAX5, PLCB1, TRAPPC9
GO:0016339	calcium-dependent cell-cell adhesion via...	15	4	1.03	0.016	CDH13, CDH17, CDH19, CDH20
GO:0009755	hormone-mediated signaling pathway	87	12	5.99	0.014	AR, ASIP, CRHBP, ESR1, ESRRB, KMT2D, NR5A1, PPARG, PRLR, REN, RNF14, SIRT1
GO:0008344	adult locomotory behavior	46	7	3.17	0.006	APP, CHL1, FGF12, NR4A2, NTAN1, PCDH15, SEZ6L
GO:0007274	neuromuscular synaptic transmission	9	4	0.62	0.002	ADARB1, CHRNA1, FCHSD2, SLC5A7
GO:0007171	activation of transmembrane receptor pro...	8	3	0.55	0.014	DGKQ, NRG1, PRLR
GO:0007156	homophilic cell adhesion via plasma memb...	57	11	3.92	0.001	CDH13, CDH17, CDH19, CDH20, CDH23, CNTN4, DSCAML1, IGSF11, MYOT, PCDH15, RET
GO:0007155	cell adhesion	508	53	34.97	0.015	ADAMTS12, ADGRL3, AMIGO3, ASS1, ASTN2, CDH13, CDH17, CDH19, CDH20, CDH23, CHL1, CLDN23, CNTN4, COL8A1, CTNNA1, CTNNA3, DSCAML1, DTX1, DYSF, EGFLAM, EPHA8, GPC4, IGSF11, ITGA11, ITGB2, LAMA3, LSAMP, MACF1, MAD1L1, MEGF9, MYH9, MYOT, NCAM1, NEGR1, NFKBIZ, NRXN1, PARD3B, PARVA, PCDH15, PLXNA1, PLXNA2, PLXNA4, PPP3CA, RDX, RET, STAB2, STK10, TLN1, TMEFF2, TNC, VTN, VWF, ZDHHC2
GO:0003073	regulation of systemic arterial blood pr...	39	6	2.68	0.003	ADRA1D, ADRB2, AR, NCALD, REN, WNK1
GO:0001967	suckling behavior	7	3	0.48	0.009	APP, CNTFR, UBE2Q1
GO:0001778	plasma membrane repair	7	3	0.48	0.009	DYSF, MYH9, SYT11

SUPPLEMENTARY FIGURES

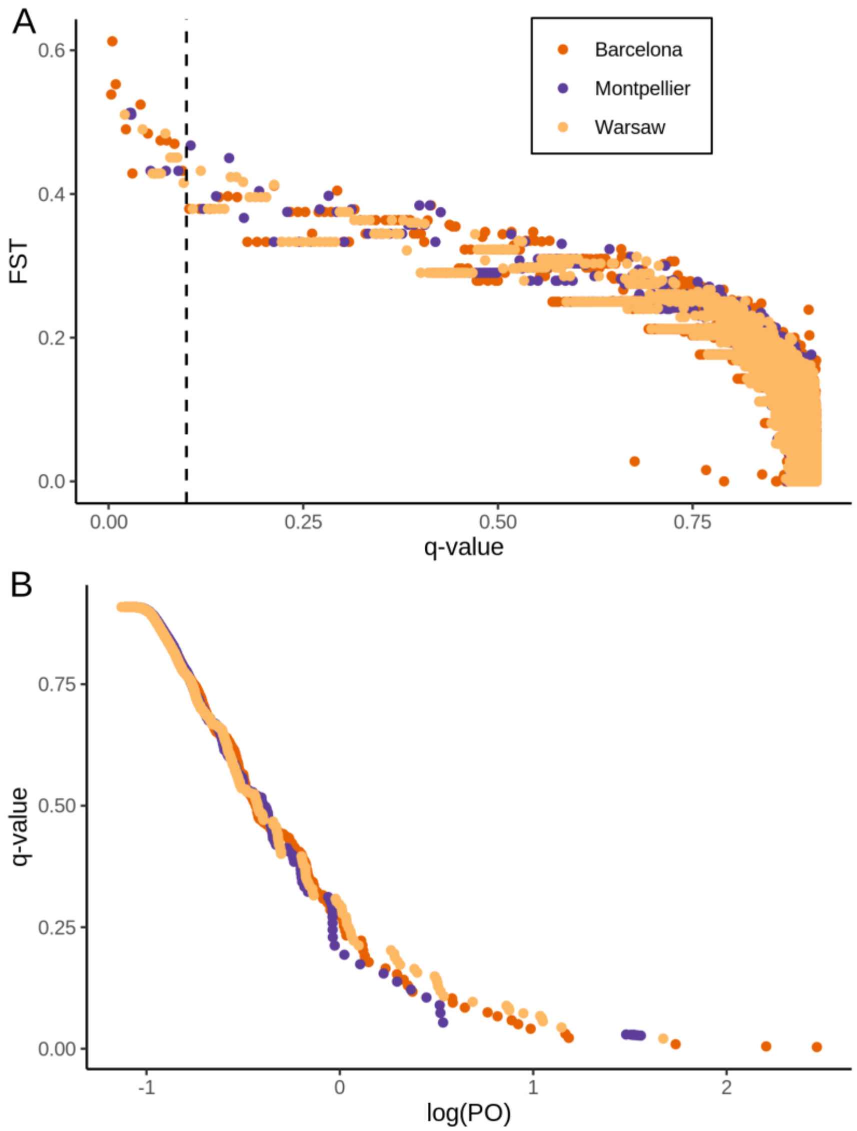

Figure S1: Results of the Bayescan tests between forest and urban great tits, for each of the three urban-forest pairs. (a) Distribution of F_{ST} values per position in function of q-value. (b) Distribution of q-value in function of log(PO).

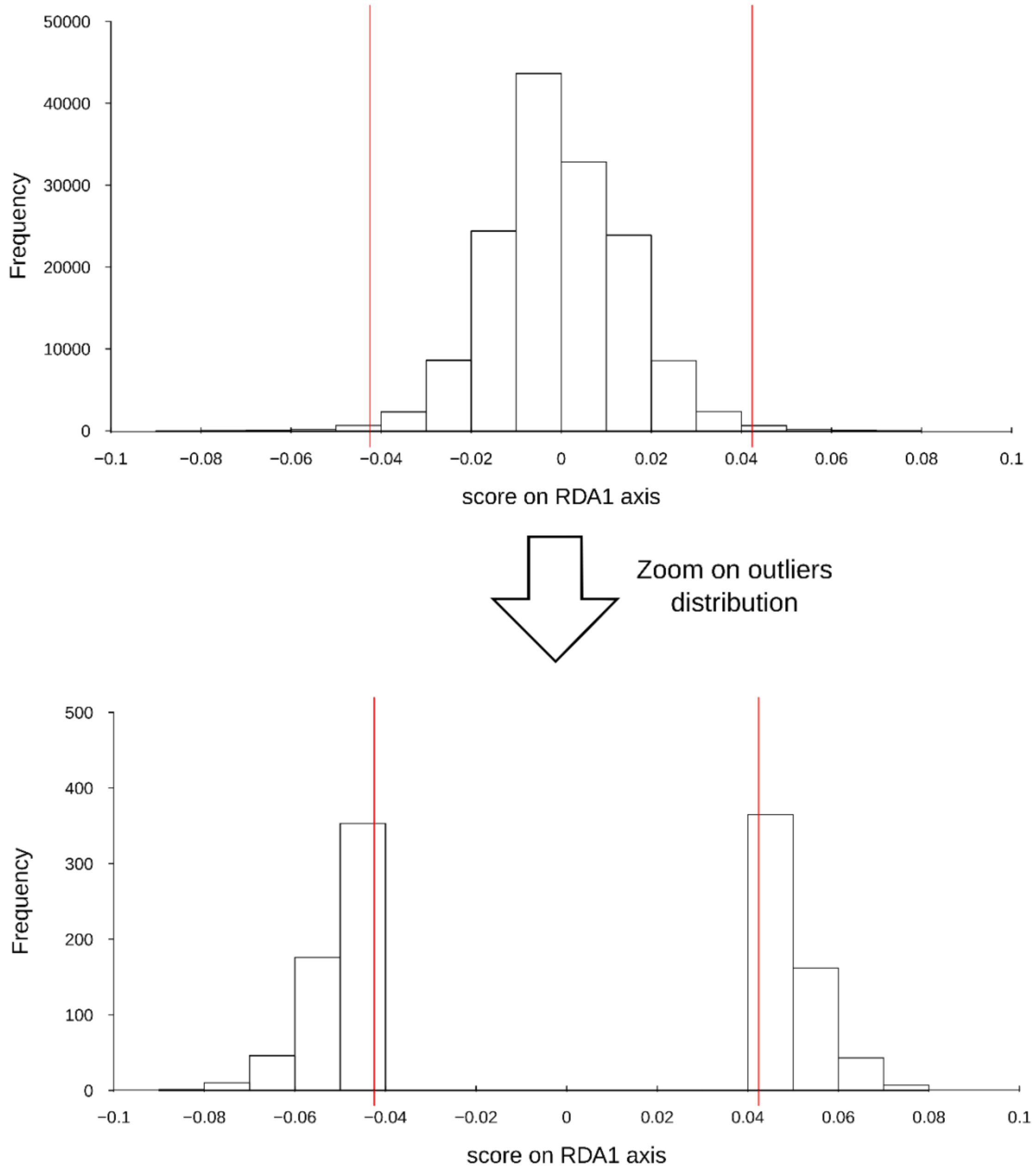

Figure S2: Distribution of genomic RDA loadings of each locus (above), and outliers (below). Vertical red lines represent the thresholds used to select outliers (please see methods for more details).

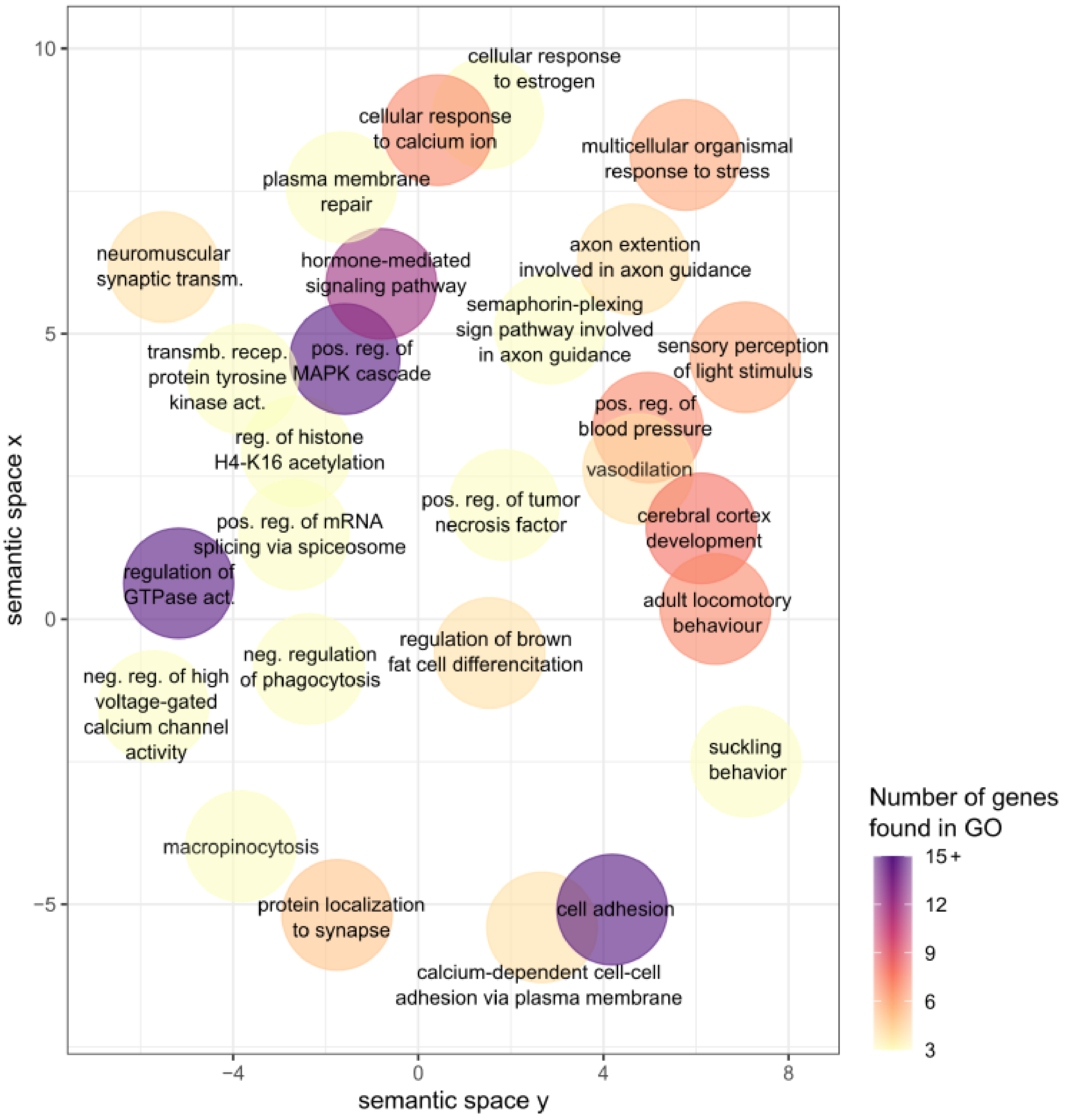

<u>Figure S3:</u> Semantic space representation for enriched GO terms from genes associated with genetic outlier (the outliers of the RDA analyses). Semantic space coordinates have been calculated based on similarity of GO term word composition using REVIGO. The color gradient indicates the number of genes involved in each GO term.

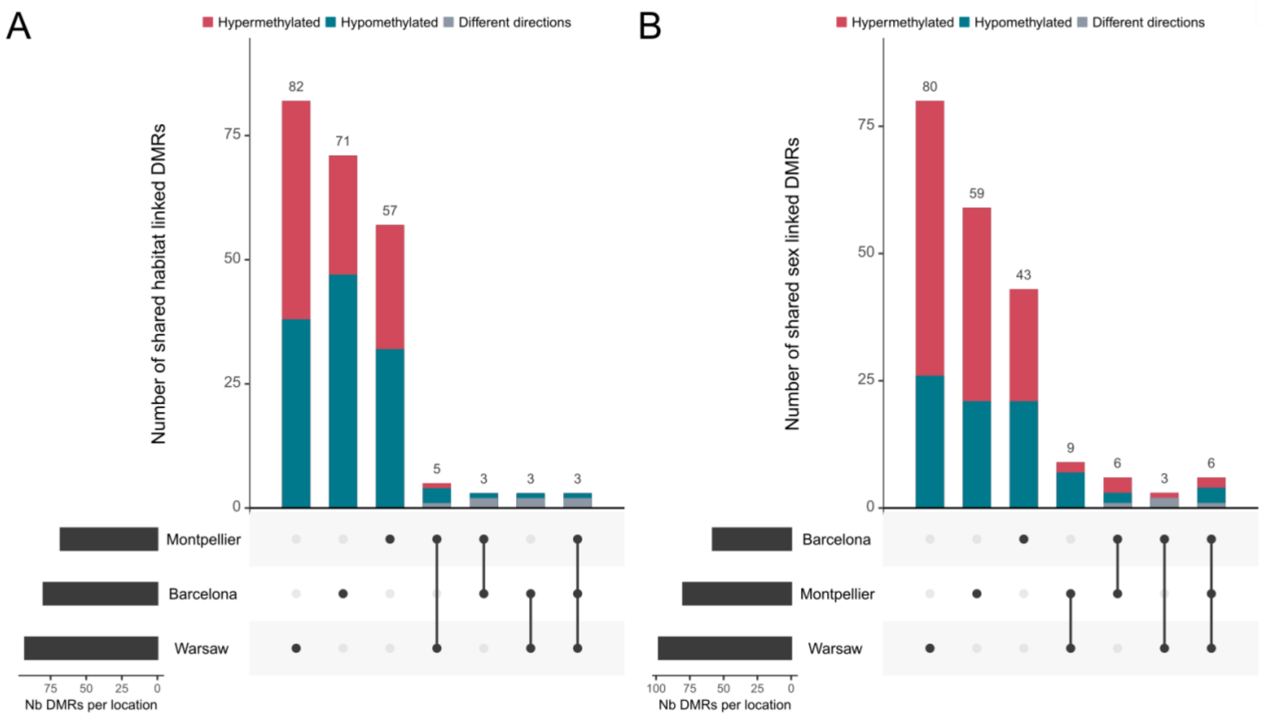

<u>Figure S4:</u> Sharing among each location of the significant DMRs found between a) forest and urban habitats, and b) females and males great tits. Hypermethylated DMRs a) in urban, b) in males) are shown in red, hypomethylated in blue and for shared DMRs, grey represent cases where a DMRs was found in multiple locations but for which the direction of methylation was opposite.

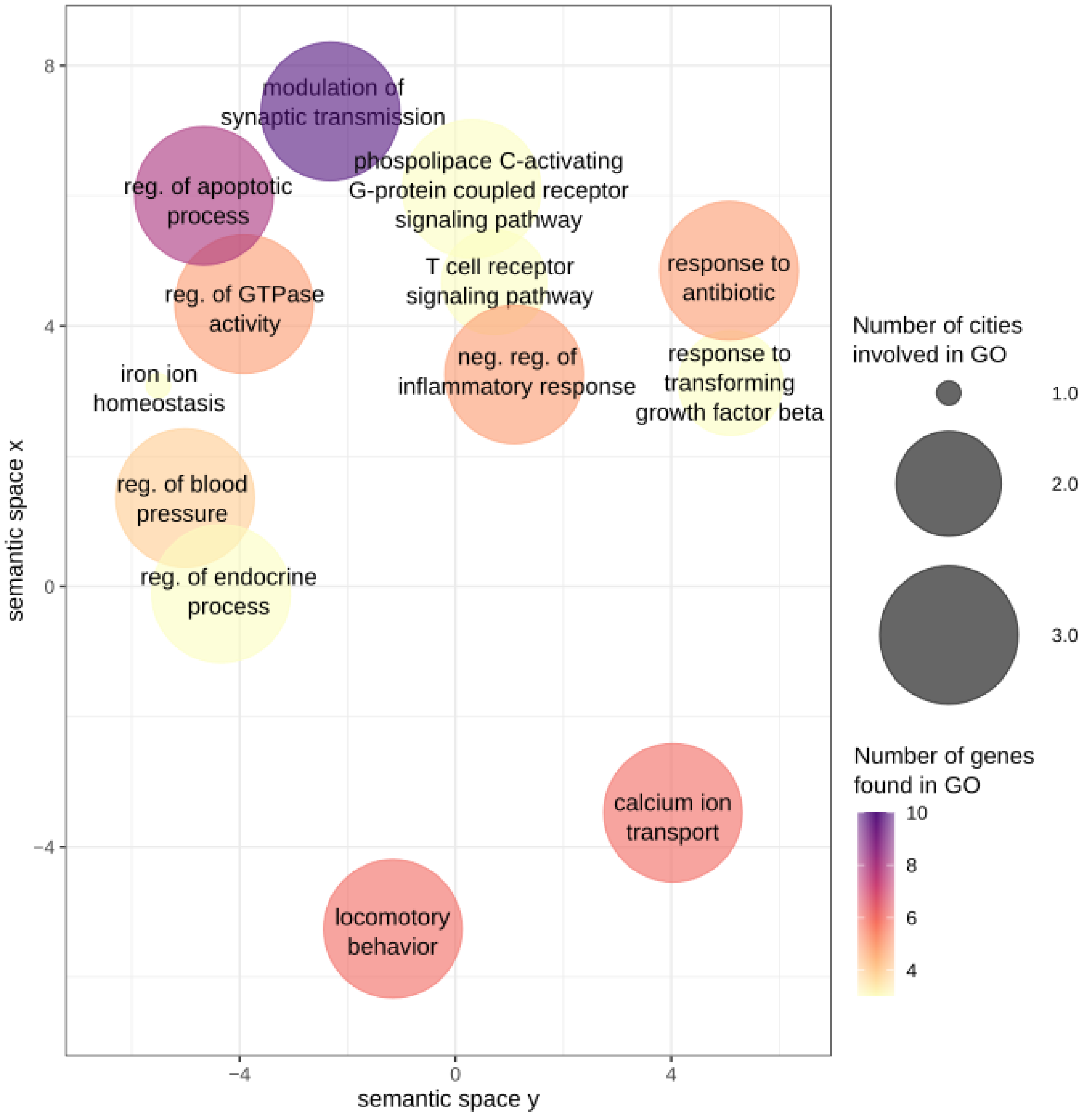

<u>Figure S5:</u> Semantic space of enriched GO terms from genes associated with urbanization-linked DMRs. Semantic space coordinates have been calculated based on similarity of GO term word composition using REVIGO. The color gradient indicates the number of genes involved in each GO term and circle size indicates the number of city contributing to each category.

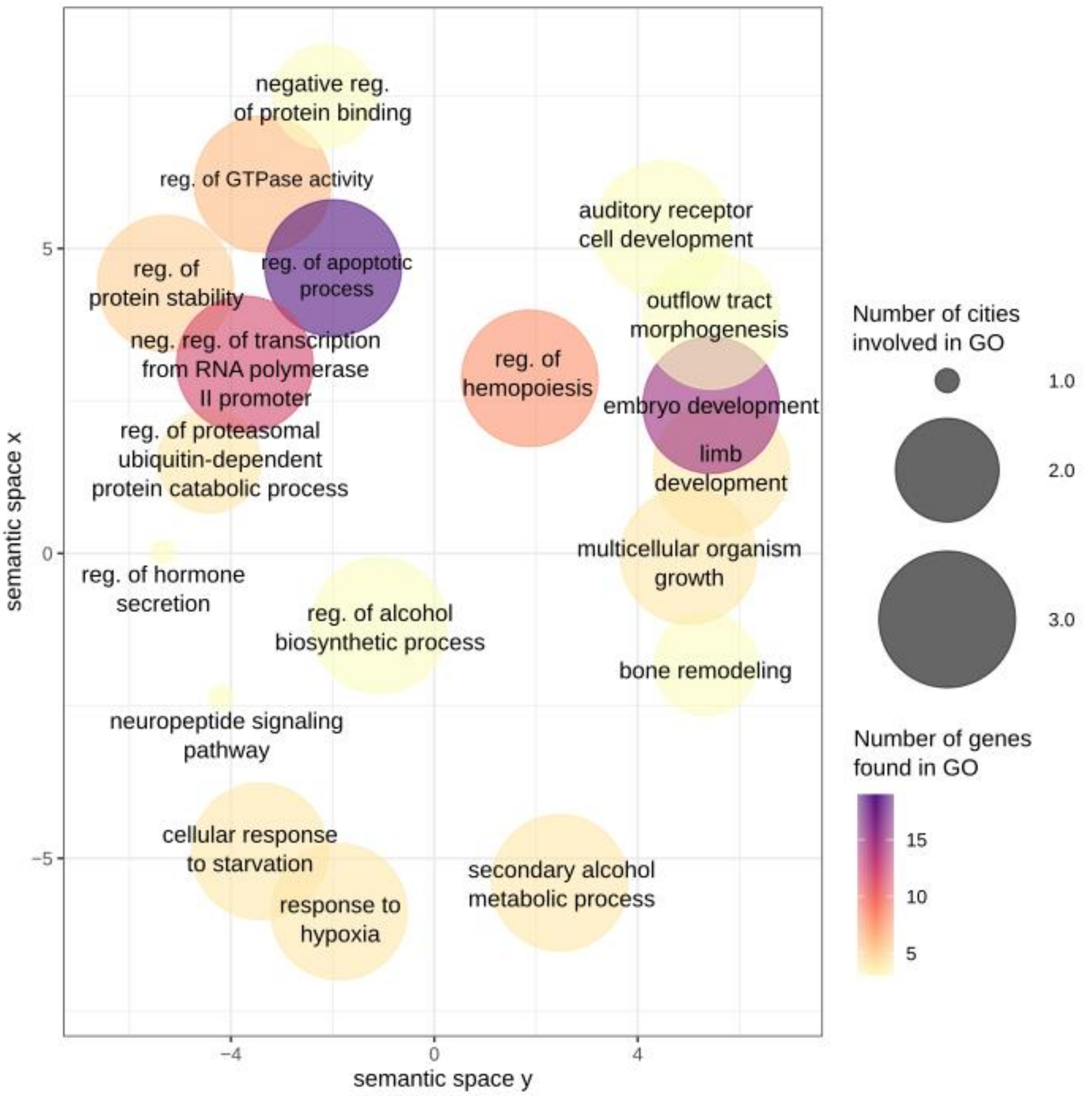

Figure S6: Semantic space of enriched GO terms from genes associated with sex-linked DMRs. Semantic space coordinates are calculated based on similarity of GO term word composition using REVIGO. The color gradient indicates the number of genes involved in each GO term and circle size indicates the number of city contributing to each category.

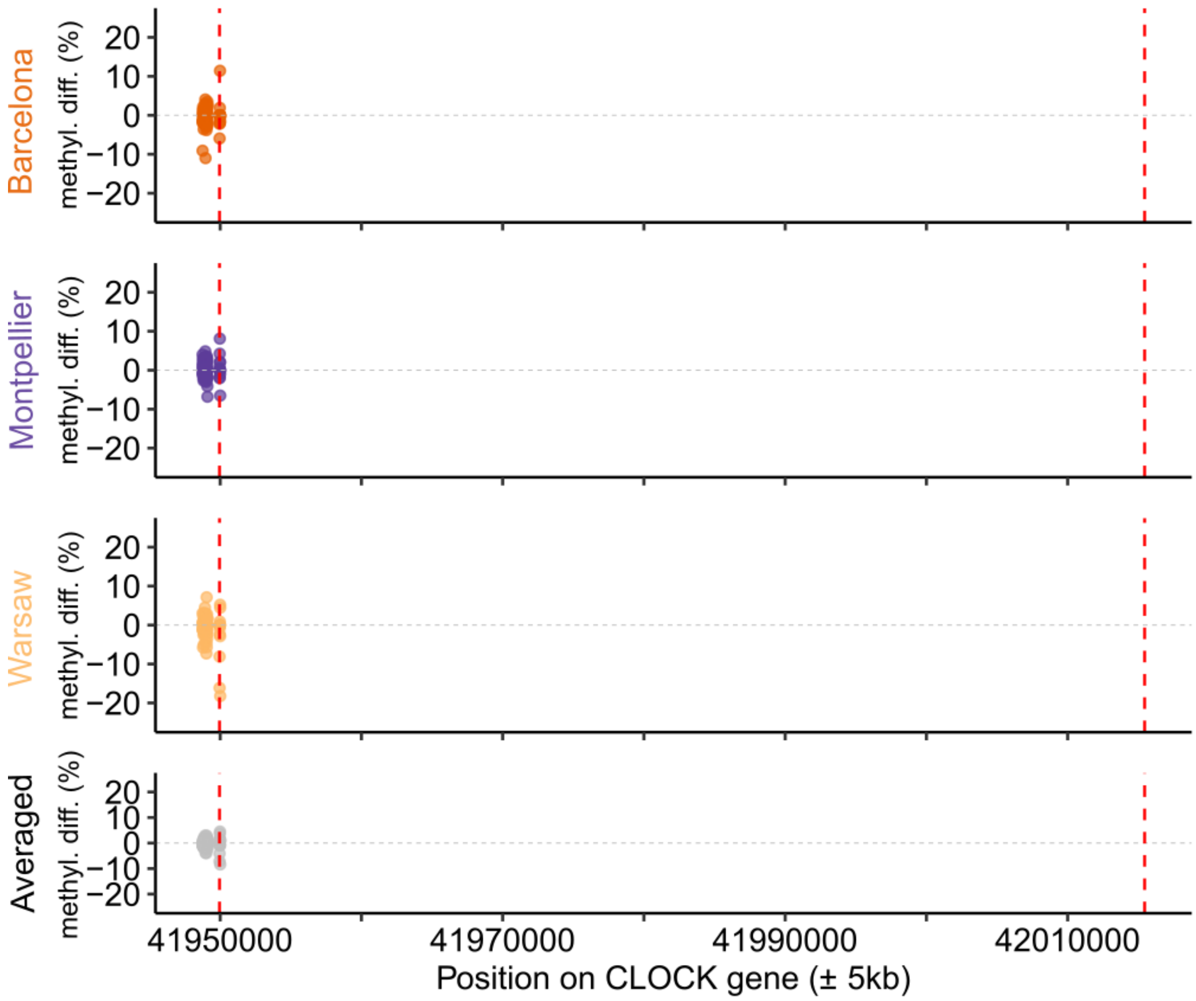

<u>Figure S7:</u> Mean methylation difference per base along the CLOCK gene. Vertical dashed lines materialised start and end of the gene.

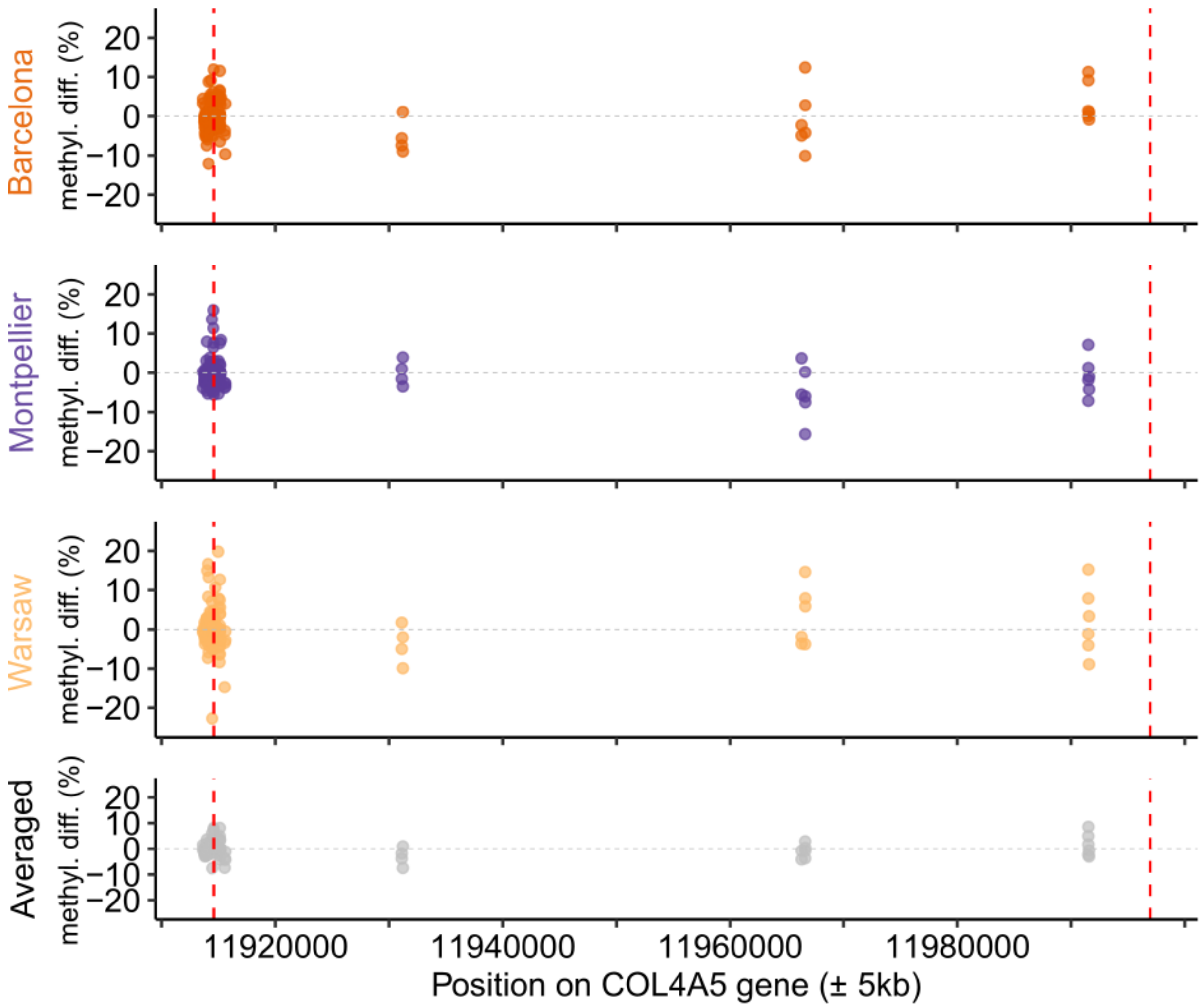

<u>Figure S8:</u> Mean methylation difference per base along the COL4A5 gene. Vertical dashed lines materialised start and end of the gene.

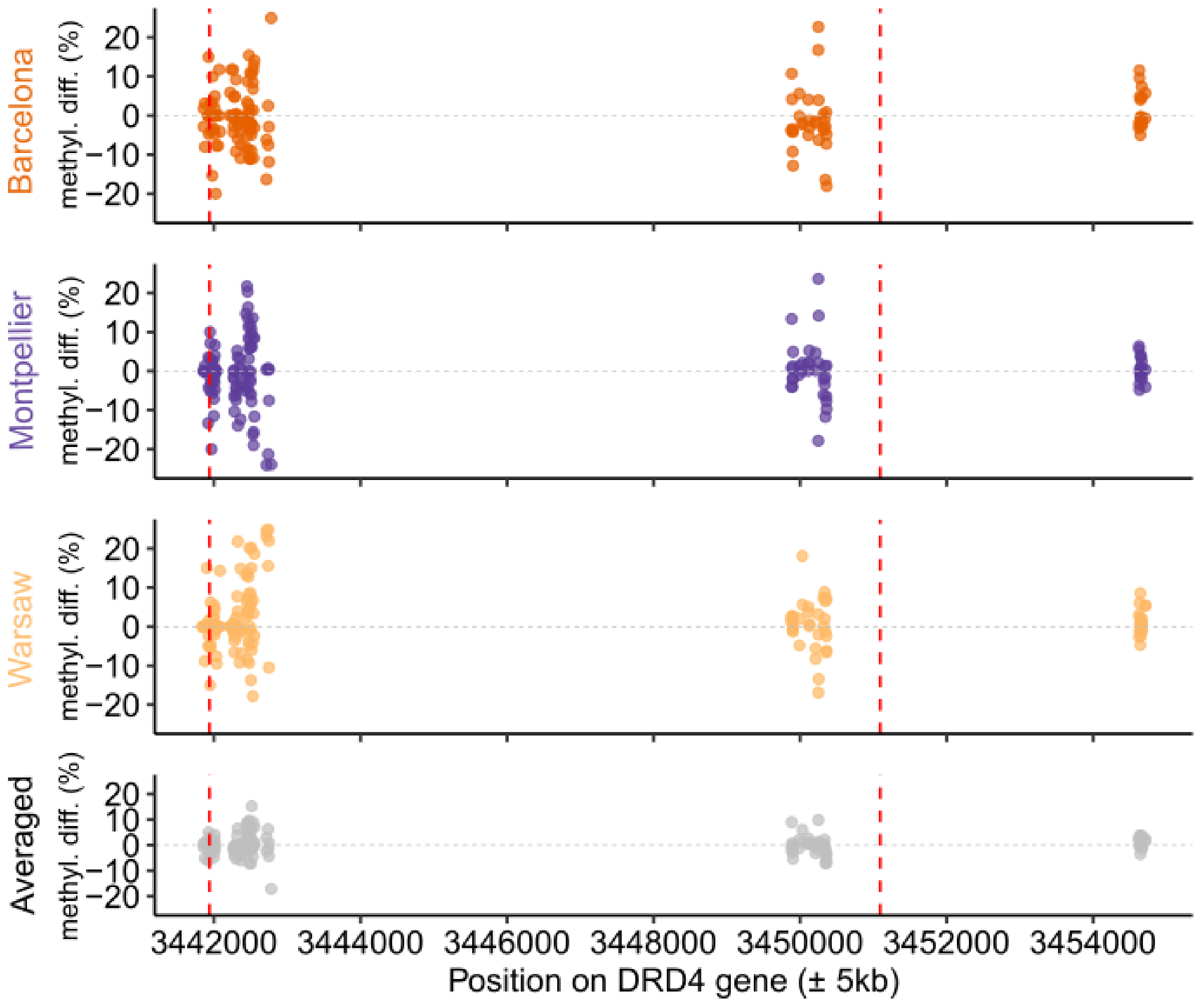

<u>Figure S9:</u> Mean methylation difference per base along the DRD4 gene. Vertical dashed lines materialised start and end of the gene.

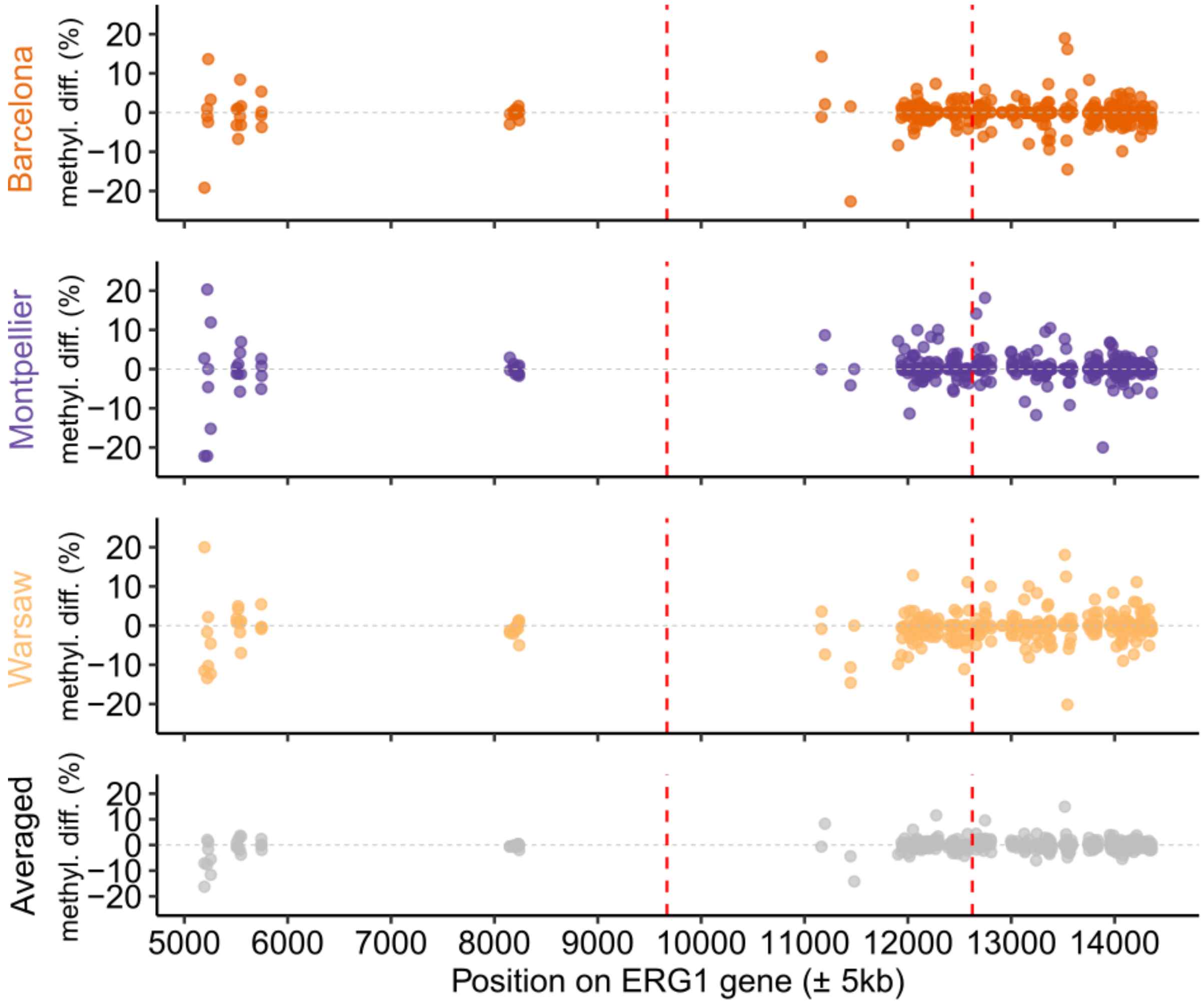

Figure S10: Mean methylation difference per base along the EGR1 gene. Vertical dashed lines materialised start and end of the gene.

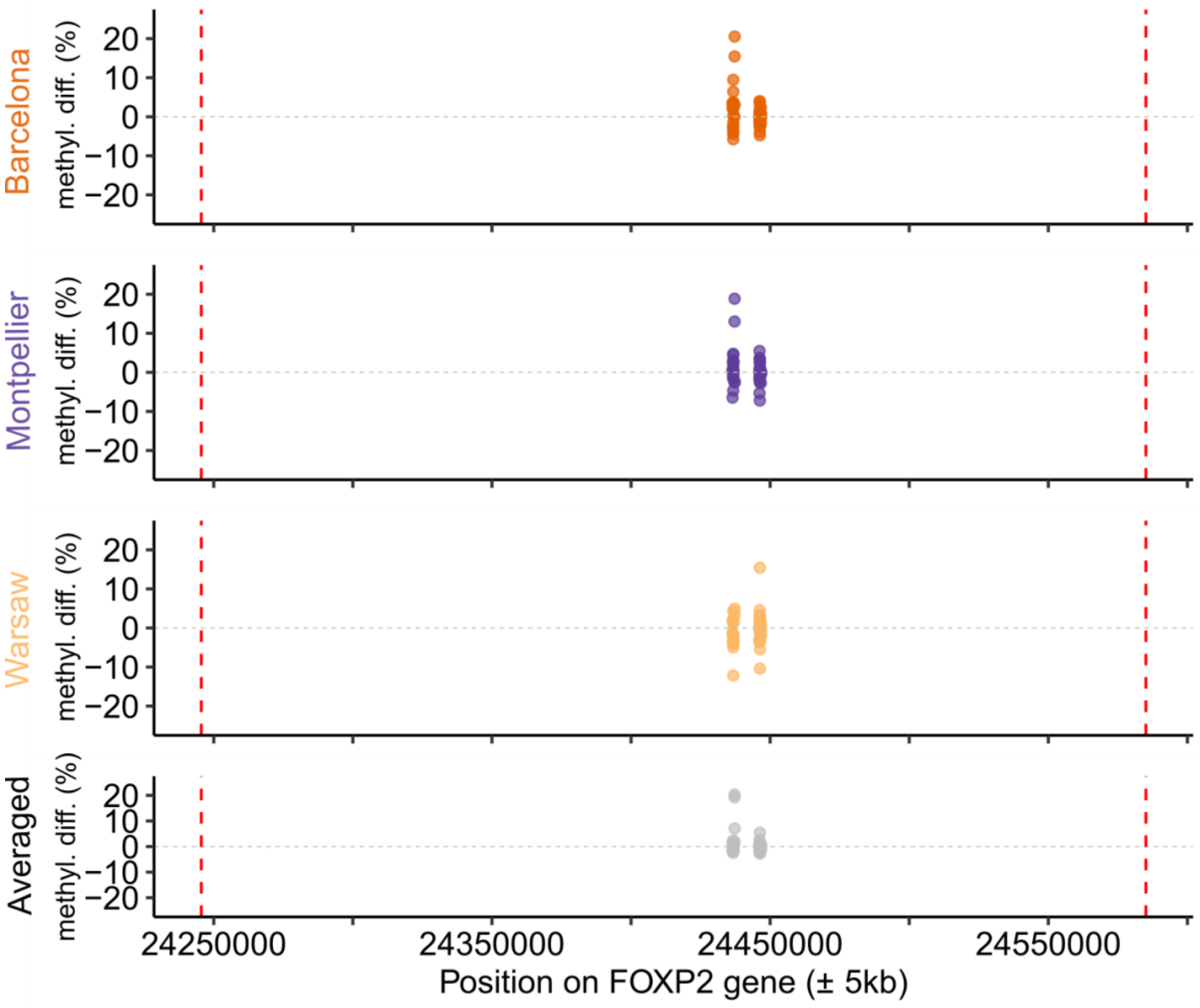

<u>Figure S11:</u> Mean methylation difference per base along the FOXP2 gene. Vertical dashed lines materialised start and end of the gene.

SUPPLEMENTARY BIBLIOGRAPHY

Bosse, M., Spurgin, L. G., Laine, V. N., Cole, E. F., Firth, J. A., Gienapp, P., … Slate, J. (2017). Recent natural selection causes adaptive evolution of an avian polygenic trait. *Science*, *358*(6361), 365–368. https://doi.org/10.1126/SCIENCE.AAL3298/SUPPL_FILE/AAL3298_BOSSE_DATABASE-S1.XLSX

Laine, V. N., Gossmann, T. I., Schachtschneider, K. M., Garroway, C. J., Madsen, O., Verhoeven, K. J. F., … Groenen, M. A. M. (2016). Evolutionary signals of selection on cognition from the great tit genome and methylome. *Nature Communications*, *7*, 10474. https://doi.org/10.1038/ncomms10474

Lindner, M., Laine, V. N., Verhagen, I., Viitaniemi, H. M., Visser, M. E., van Oers, K., & Husby, A. (2021). Rapid changes in DNA methylation associated with the initiation of reproduction in a small songbird. *Molecular Ecology*, mec.15803. https://doi.org/10.1111/mec.15803

Mueller, J. C., Partecke, J., Hatchwell, B. J., Gaston, K. J., & Evans, K. L. (2013). Candidate gene polymorphisms for behavioural adaptations during urbanization in blackbirds. *Molecular Ecology*, *22*(13), 3629–3637. https://doi.org/10.1111/mec.12288

Riyahi, S., Sánchez-Delgado, M., Calafell, F., Monk, D., & Senar, J. C. (2015). Combined epigenetic and intraspecific variation of the *DRD4* and *SERT* genes influence novelty seeking behavior in great tit *Parus major*. *Epigenetics*, *10*(6), 516–525. https://doi.org/10.1080/15592294.2015.1046027

Steinmeyer, C., Mueller, J. C., & Kempenaers, B. (2009). Search for informative polymorphisms in candidate genes: Clock genes and circadian behaviour in blue tits. *Genetica*, *136*(1), 109–117. https://doi.org/10.1007/S10709-008-9318-Y/TABLES/4
